# Protocol for an embedded randomised controlled trial of Early versus Late Stopping of Antibiotics in children with Febrile Neutropenia (ELSA-FN)

**DOI:** 10.1371/journal.pone.0311523

**Published:** 2024-12-09

**Authors:** Coen Butters, Anneke Grobler, Alannah Rudkin, Li-yin Goh, Heather Werdenburg, Diane Hanna, Theresa Cole, Jim Buttery, Karin Thursky, Andrew Davidson, Gabrielle M. Haeusler

**Affiliations:** 1 Department of General Paediatrics and Adolescent Medicine, John Hunter Children’s Hospital, Newcastle, Australia; 2 Infection, Immunity and Global Health, Murdoch Children’s Research Institute, Parkville, Australia; 3 Department of Paediatrics, The University of Melbourne, Parkville, Australia; 4 Murdoch Children’s Research Institute, Parkville, Australia; 5 Centre for Health Analytics, Melbourne Children’s Campus, Parkville, Australia; 6 Melbourne Children’s Trials Centre, Murdoch Children’s Research Institute, Parkville, Australia; 7 Children’s Cancer Centre, Royal Children’s Hospital, Parkville, Australia; 8 Allergy and Immunology, Royal Children’s Hospital, Parkville, Australia; 9 Infectious Diseases Unit, Royal Children’s Hospital, Parkville, Australia; 10 Health Informatics Group and SAEFVIC, Murdoch Children’s Research Institute, Parkville, Australia; 11 Department of Infectious Diseases, Peter MacCallum Cancer Centre, Parkville, Australia; 12 National Centre for Antimicrobial Stewardship, Department of Infectious Diseases, The University of Melbourne, Parkville, Australia; 13 Department of Medicine, The University of Melbourne, Parkville, Australia; 14 Department of Anaesthesia, Royal Children’s Hospital, Parkville, Australia; 15 Department of Critical Care, The University of Melbourne, Parkville, Australia; National Defense Medical Center, TAIWAN

## Abstract

In children with cancer, febrile neutropenia (FN) is one of the most common complications of treatment, a leading cause of unplanned and prolonged hospital admission and is the key driver of antibiotic exposure. Co-designed with key stakeholders, ‘Early versus Late Stopping of Antibiotics in high-risk FN’ (*ELSA-FN*) is a randomised controlled, non-inferiority trial that compares stopping antibiotics in clinically stable patients after 48 hours with the current standard of care, continuing antibiotics until absolute neutrophil recovery. As an Australian first, we will exploit the potential of electronic medical record (EMR) systems, embedding all key aspects of the trial including screening, consent, randomisation and data collection into standard clinical and EMR workflows. We aim to randomise 320 children with high-risk FN and prospectively collect data on safety, acceptability to clinicians and families, as well as several secondary outcomes related to antibiotic exposure. The findings will contribute to optimal antibiotic use in children with FN internationally and inform design and implementation of future EMR-embedded trials.

## Introduction

### Background and rationale

Febrile neutropenia (FN) is one of the most common complications of childhood cancer treatment and a leading cause of unplanned and prolonged hospital admission [1]. While advances in treatment and supportive care have resulted in 5-year survival of over 90% for most paediatric haematological malignancies, the approach to FN management remains unchallenged [2]. Since the 1970’s, the standard of care has been intravenous antibiotics commenced at the onset of fever and continued, even in the absence of documented bacterial infection, until resolution of neutropenia. This remains the standard of care at many centres, despite low rates of serious bacterial infection and infection-related mortality [1, 3, 4]. International recommendations vary widely (Table 1), which reflects the paucity of randomised control trial (RCT)-level data, particularly for high-risk FN (expected absolute neutrophil count (ANC) <500 cells/mm^3^ for ≥7 days). This translates to repeated and protracted antibiotic exposure throughout cancer treatment.

**Table 1:** Factors informing empiric antibiotic duration in FN according to current international recommendations.

Reference	Negative blood culture	Afebrile for 24 hour	Afebrile for 48 hours	“Haemodynamic stability”	“Response to treatment”	ANC >500 cells/mm^3^	Evidence of bone marrow recovery	Minimum antibiotic duration (hours)	Comments
IDSA, 2010 [5]	•					•	•		No specific recommendation for children or high-risk FN
ESMO, 2016 [6]	•		•			•		48	Recommends considering stopping antibiotics in low-risk patients who have been afebrile for 5–7 days (irrespective of ANC)
8th ECIL, 2020 [7]	•	•		•				*72	*Low-risk recommendation only
NICE, 2020 [8]	•				•				Cease after “response to treatment” (irrespective of ANC)
International Pediatric FN Guideline, 2023 [9]	•	•					•	48	Confirms 2017 recommendation to continue in high- and low-risk patients until evidence of bone marrow recovery

IDSA, Infectious Diseases Society of America; ESMO, European Society of Medical Oncology; ECIL, European Conference on Infections in Leukaemia; NICE, National Institute for Health and Care Excellence

Antibiotics in children with cancer have a range of immediate adverse effects such as nausea, vomiting, nephrotoxicity, electrolyte disturbance, hypersensitivity reactions and added myelosuppression [10]. For many children, this translates to decreased quality life in addition to the significant financial costs for families and the healthcare system [11–13]. Prior and prolonged antibiotic exposure is also a risk factor for long term adverse effects, including infection with antimicrobial resistant (AMR) bacteria, increased rates of invasive candidiasis and infection with *Clostridioides difficile* [10]. Those that develop AMR infections have a poorer outcome, including an increased risk of death [14–17]. Furthermore, there is emerging evidence regarding the broad effects of antibiotics on the gut microbiota and the potential long-term implications for children with cancer [10]. Antibiotic exposure is associated with graft-versus-host disease (GVHD) in patients undergoing haematopoietic cell transplant (HCT) and, in the case of immune checkpoint inhibitors, prior antibiotic use is associated with worse treatment response and decreased overall survival [10, 18].

Although there are eight RCTs in FN that have compared prolonged (i.e. continue until ANC recovery) with short course (i.e. discontinue irrespective of ANC) antibiotic therapy in FN [19], only one study focused on the high-risk patient population (acute leukaemia or HCT) and this study excluded children [20]. Despite these limitations, short course antibiotics have been reported to reduce antibiotic exposure without an increase in adverse events or bacterial infection [19]. Of the three paediatric-specific, only one (n = 176) included high-risk FN episodes (n = 88, 50%). This study found that in patients with FN and a confirmed respiratory virus, stopping antibiotics at 48 hours was not associated with higher rates of clinical failure compared to standard of care [21].

The Early versus Late Stopping of Antibiotics in Febrile Neutropenia (*ELSA-FN*) trial is the first clinical trial to prospectively address the optimal duration of antibiotics in children with cancer and high-risk FN. *ELSA-FN* will embed a RCT in the electronic medical record (EMR) to address a critical research gap and inform clinical practice. We will also evaluate the acceptability of this intervention to healthcare providers, patients and families and its cost-effectiveness relative to current practice (*ELSA-Impact*).

While RCTs are the foundation of substantial improvements in survival of children with cancer, cost and resource use are major barriers to their conduct [22, 23]. Novel and pragmatic approaches to RCTs are required to ensure equitable access, sustainability and efficiency within the overburdened healthcare system [24]. Embedded clinical trials, like *ELSA-FN*, utilise existing clinical infrastructure and staff, take place in the clinical setting and harness data collected through routine clinical care [25]. Embedding RCTs within the EMR could improve recruitment and significantly reduce costs when compared to conventional trial design [26]. Throughout the process of embedding patient recruitment, randomisation, intervention and data collection in clinical workflows and within the EMR system, we aim to study in real-time the barriers, enablers and cost-effectiveness of embedded trials methodology (*ELSA-EMR*).

### Objectives

The primary objective of *ELSA-FN* is to determine if stopping antibiotics in clinically stable children with high-risk FN without documented bacterial infection before ANC ≥500 cells/mm^3^ (Stop arm) is non-inferior in terms of safety measures compared to continuing antibiotics until ANC recovery (Standard of Care (SOC) arm).

The secondary objectives are:

1. *ELSA-Impact*: To determine if stopping antibiotics prior to ANC recovery is acceptable to healthcare providers, patients and families, and is cost effective.
2. *ELSA-EMR*: To identify barriers, enablers and the cost-effectiveness of EMR embedded trials.

#### ELSA-FN methods: Participants, interventions, and outcomes

This is a randomised, controlled non-inferiority trial with patient enrolment planned at the Royal Children’s Hospital Melbourne, the largest tertiary paediatric hospital in Australia. The study protocol has been prepared according to the SPIRIT checklist for randomised controlled trials and is registered with Clinicaltrials.gov (NCT04948463) [27, 28].

### Research ethics approval

The clinical trial protocol is approved by the Human Research Ethics Committee of the Royal Children’s Hospital, Parkville (HREC 74690). Informed consent will be required to participate in all aspects of *ELSA-FN* and by participants in *ELSA-Impact* focus groups. Any amendment will be reviewed by the Human Research Ethics Committee prior to implementation. Changes to the protocol will be communicated to clinicians in writing and during quarterly meetings with the clinical oncology group.

### ELSA-FN trial design

*ELSA-FN* was co-designed through regular meetings with key stakeholders; oncology and infectious diseases physicians and nurses, junior medical officers and parents of children with cancer. *ELSA-FN* was designed to be pragmatic and recruit a high-risk population with outcomes routinely recorded in the electronic medical record (Epic EMR System). The trial population and outcome measures were defined and translated to EMR definitions or digital phenotypes. These digital phenotypes (S1 Table) were tested and refined through repeat cycles of improvement [29, 30].

### ELSA-FN eligibility criteria

The eligible trial population are children admitted to the Royal Children’s Hospital Melbourne with high-risk FN (Table 2). High-risk FN is defined as leukaemia and lymphoma in dose-intensive treatment phases or patients with any diagnosis who are undergoing HCT. Patients must be clinically stable, afebrile and have no clinical or microbiological evidence of bacterial infection to be randomised. Patients with prolonged fever (≥5 days) will often have investigation for invasive fungal disease and randomisation to short-course antibiotics would not be appropriate. Patients can be enrolled and randomised in the study more than once, with each considered a discrete episode of FN. A new FN episode is defined as a new fever occurring during a new episode of neutropenia (ANC <500 cells/mm^3^) and at least 28 days after last randomisation.

**Table 2:** ELSA-FN- trial inclusion and exclusion criteria.

Inclusion criteria	Exclusion criteria
• Age <18y • Diagnosis of: a) Acute myeloid leukaemia (AML); or b) Acute lymphoblastic leukaemia (ALL) in chemotherapy-intensive phases of induction, re-induction, intensification or consolidation; or c) Lymphoma receiving intensive chemotherapy as per St. Jude Children’s Research Hospital Total Therapy XVII (Total 17) protocol; or d) Any disease within 100 days of allogeneic or autologous HCT • Absolute neutrophil count <500 cells/mm^3^ • Commenced empiric FN antibiotics (any of: piperacillin-tazobactam, cefepime, ceftazidime, ciprofloxacin, +/-vancomycin, +/- amikacin, +/- gentamicin) • At least one temperature measured by axillary or tympanic thermometer (≥38.0°C) and afebrile (temperature <38.0°C) for ≥48 hours but no more than 96 hours • Clinical stability for ≥48 hours (conscious state, respiratory rate, blood pressure, or oxygen saturations not breaching mandatory Medical Emergency Team call criteria OR heart rate in the clinical review criteria [>95^th^ percentile for age] in 48 hours prior to randomisation) [31]	• Microbiologically defined bloodstream infection: Documented positive blood culture since onset of FN episode • Clinically or microbiologically defined infection: documented other infection requiring antibiotic treatment (eg. urinary tract infection) • Admitted to the Intensive Care Unit (ICU) • Prolonged fever (documented daily temperature ≥38.0°C for ≥5 days) • Within 28 days of last randomisation

### ELSA-FN sample size

Retrospective local data indicates that 30% of patients with high-risk FN will have recurrence of fever. Focus groups with clinicians indicated that it would be clinically acceptable for up to 45% of those in the intervention arm to have recurrence of fever. Thus, the sample size was calculated with a non-inferiority margin of 15% between the intervention and comparison arms.

If each group includes 147 participants, a two-group large-sample normal approximation test of proportions with a one-sided 2.5% significance level will have 80% power to reject the null hypothesis, assuming that the expected difference in proportions is 0 and the proportion in the standard group is 0.3. This sample size assumes that all participants are independent, however, some participants can be enrolled in the trial more than once. This can be accommodated in the sample size calculation by a design effect. We calculated the design effect to be 1.06 using the individual randomisation and independent working correlation formula in Yelland *et al* [32]. This assumes that 50% of enrolments will be enrolled only once and that the proportion in the intervention group would be 0.3 and in the comparison group would be 0.3 to 0.33. The correlation (ρ) is assumed to be 0.3. The design effect was applied to the calculated sample size, with a final sample size of approximately 160 per arm, that is 320 patient-episodes in total. We plan an interim review by an independent statistician after the recruitment of approximately 100 patients to assess the adequacy of the sample size.

### ELSA-FN recruitment

The projected recruitment period is three years. All patients with eligible diagnosis that are (i) admitted for neutropenic observation; (ii) admitted to inpatient ward with FN or (iii) develop FN while an inpatient will generate a Best Practice Advisory (BPA) alert to treating medical or pharmacy clinicians.

### ELSA-FN intervention and control

**Fig 1:**
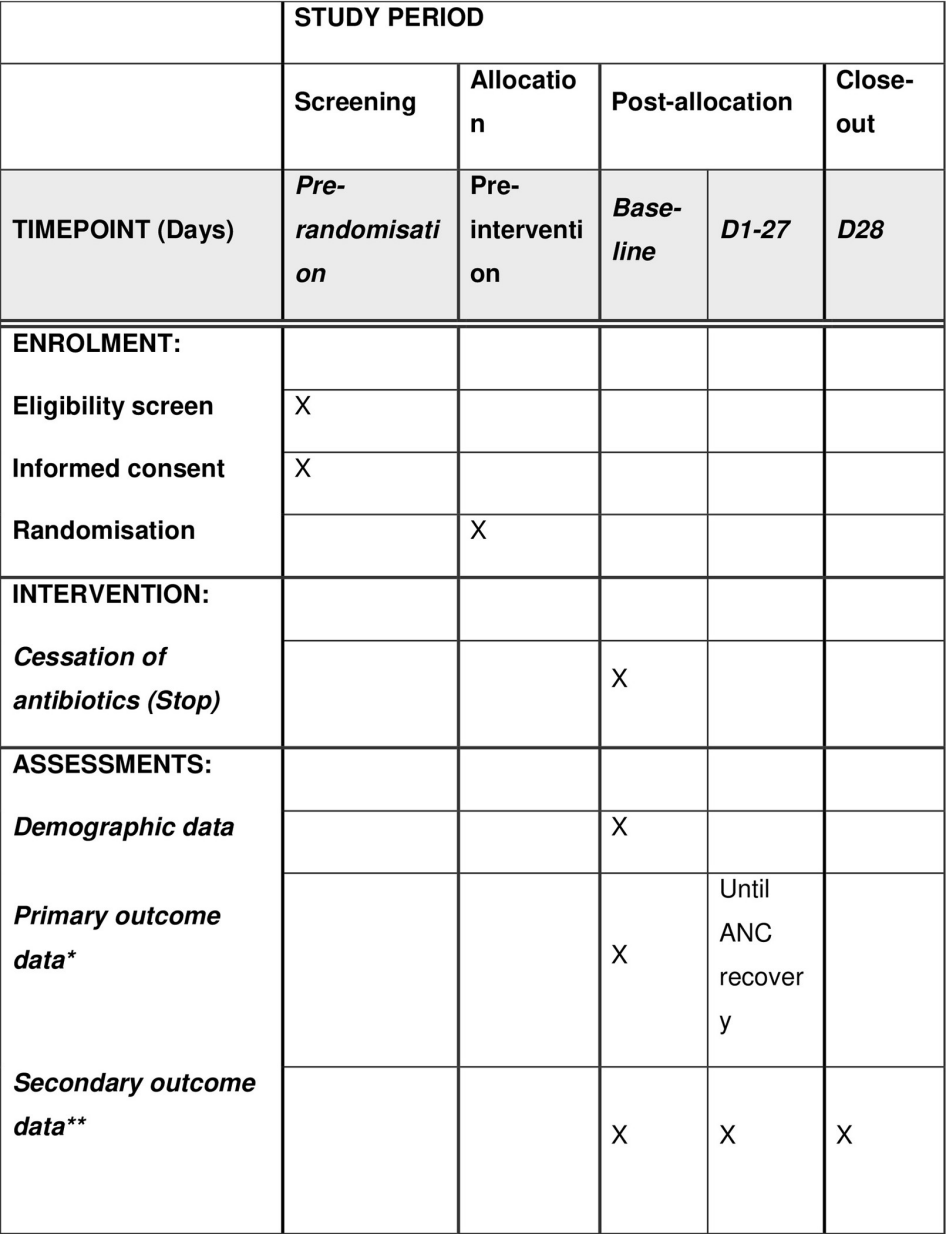
SPIRIT schedule of enrolment, intervention, and assessment for ELSA-FA [27].

Children with high-risk FN will be randomly assigned to the intervention or control arms and followed up for 28 days (Fig 1).

- Intervention (Stop): Stopping empiric FN antibiotics after resolution of fever for 48 hours, irrespective of absolute neutrophil count.
- Control (SOC): Continuing empiric FN antibiotics after resolution of fever for 48 hours and until recovery of ANC, as defined by the treating clinician (usually ANC ≥200–500 cells/mm^3^).

### ELSA-FN primary and secondary outcomes

The primary outcome is ‘unfavourable clinical course’ defined as any of the following occurring during the same period of severe neutropenia (ANC <500 cells/mm^3^):

- Recurrence of temperature ≥38 degrees Celsius (i.e. new fever episode after afebrile period of at least 48 hours)
- Clinical instability (one or more of conscious state, respiratory rate, blood pressure, heart rate, oxygen saturation meeting mandatory emergency call criteria OR two or more respiratory rate, blood pressure, heart rate or oxygen saturations simultaneously (+/- 4h) meeting clinical review criteria)
- Admission to the ICU
- New positive blood culture collected after randomisation (with any organism)
- Death

*ELSA-FN* will also measure secondary outcomes up to day 28 post-randomisation:

- Admission to the ICU and type of organ support during the same period of neutropenia and/or 28 days of randomisation
- New infection after randomisation and during the same period of neutropenia and/or 28 days of randomisation, including:
   ⚬ Microbiologically defined infection (MDI): infection that is clinically documented and microbiologically confirmed
   ⚬ Clinical documented infection (CDI): an infection that is clinically detectable, but no pathogen is identified
   ⚬ Fever without focus: Fever without a documented MDI or CDI
- All cause 28-day mortality
- Infection-related 28-day mortality (death with microbiologically proven or clinically suspected infection)
- Duration of neutropenia (measured as days from ANC <500 cells/mm^3^ to ANC ≥500 cells/mm^3^)
- Total antibiotic duration measured as length of therapy (LOT) and days of therapy (DOT), excluding antibiotic prophylaxis
- Re-commencement of any treatment antibiotic
- Total hospital length of stay in days from randomisation to hospital discharge
- Unplanned readmission to hospital inpatient ward
- *C. difficile* infection (testing as per routine clinical care)
- Antibiotic resistant colonisation or infection (testing as per routine clinical care): methicillin-resistant *Staphylococcus aureus*, Extended-Spectrum Beta-lactamase-producing *Enterobacterales*, Carbapenem-producing *Enterobacterales*, Vancomycin-resistant *Enterococcus*

**ELSA-FN methods.** *Consent process*. In ELSA-FN, an investigator or delegated member of the trial team (including clinical ward staff) will discuss the trial with the parent/legal guardian and, where appropriate, the child or adolescent. All those involved in the study will complete an online learning package that covers the trial background, key aspects of informed consent and ensures competency in the study protocol. Those completing consent are required to hold a current Good Clinical Practice certificate.

A parent/carer/participant information video has been co-designed with consumers and clinicians to supplement the digital consent process and provide visual information that is suitable across various levels of health literacy (https://www.healthanalytics.org.au/project/elsa-fn/). This will also ensure consistent information is delivered if the trial opens to recruitment at additional sites.

Consent will be completed by accessing an online Participant Information and Consent Form (PICF) and providing a digital signature. This digital consent platform has potential advantages as a real-time digital record of consent and reduces infection control issues associated with direct patient contact and traditional use of paper and pen.

**Fig 2:**
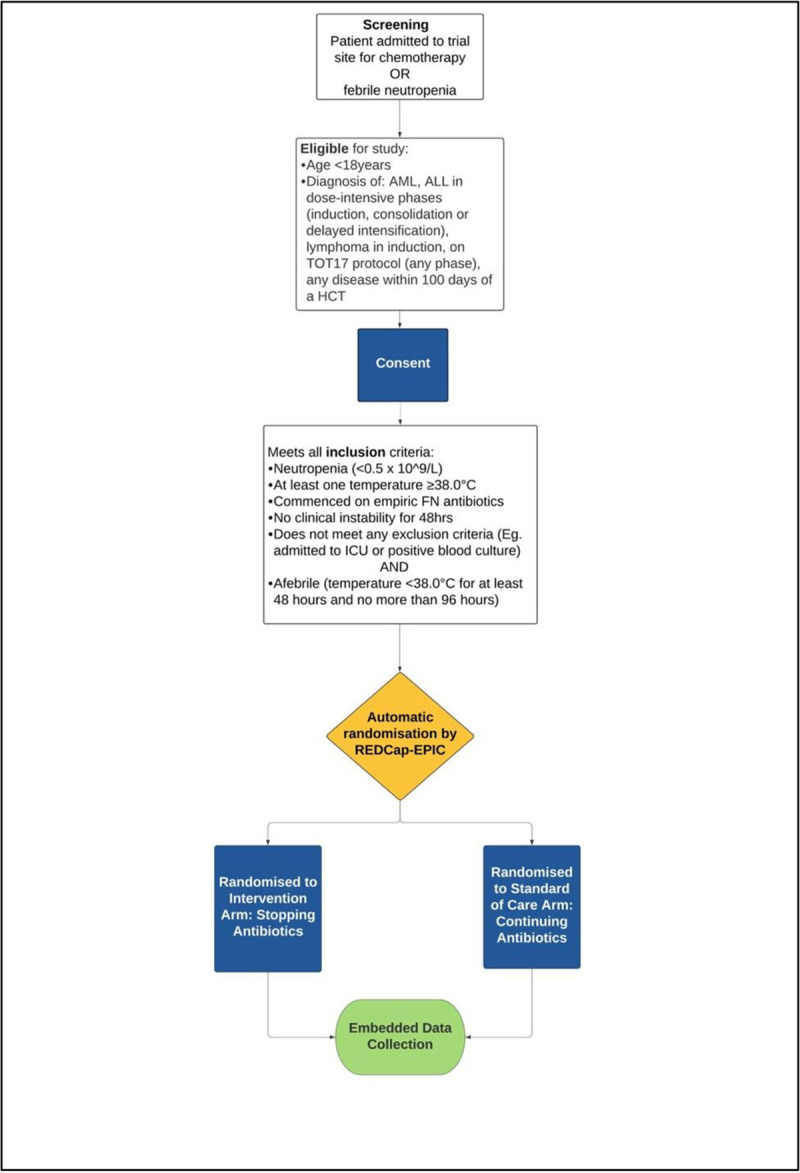
Trial overview. AML, acute myeloid leukaemia; ALL, acute lymphoblastic leukaemia; TOT17, Total therapy XVII for newly diagnosed patients with acute lymphoblastic leukaemia and lymphoma; HCT, haematopoietic cell transplant; FN, febrile neutropenia; ICU, Intensive Care Unit.

*Randomisation*, *allocation*, *sequence generation*. Following documentation of consent, patients will be enrolled and randomly assigned, in a 1:1 ratio into the two study groups (Fig 2). A statistician not directly involved in the analysis of the trial results will prepare the randomisation schedule using block randomisation. As randomisation occurs a minimum of 48 hours after FN onset, all patients will initially be managed according to local FN and sepsis care pathways. For patients meeting inclusion criteria and randomised to the intervention arm, clinicians will be prompted by a BPA notification to cease all antibiotics.

*Data collection*. Eligibility screening, enrolment, randomisation and FN data (demographic, onset/episode and outcome) will be extracted from the EMR up to day 28 post-randomisation (Fig 1). As an embedded trial, study assessments are captured in real-time while the patient is admitted to hospital. This includes recorded vital signs, clinical administration of antibiotics and all microbiology and pathology results. This routinely captured electronic patient data will form the dataset.

*Data management*. Compared with paper-based systems, the EMR has significant advantages as a data source. The data is attributable to individuals, legible (including any changes or corrections), recorded contemporaneously, and available securely through remote access [33]. Data monitoring will occur continuously by a dedicated EMR analyst. They will run regular reports on enrolled patients to ensure that the key outcome measures are being recorded with the expected quality.

The trial data, patient information and documents will be electronically stored securely for at least 15 years post-trial completion or until participating children are over 25 years of age (whichever is later). Beginning 12 months following analysis, the de-identified individual participant data will be made available long-term for use by researchers whose proposed use of the data has been ethically reviewed and approved by an independent committee.

*Statistical methods*. The incidence of the primary outcome will be calculated in each treatment arm with a 95% confidence interval. The outcome will be modelled using a generalised linear mixed model with a logit link function and a Bernoulli distribution. The model will be adjusted for baseline covariates. This model will be used to calculate the difference in proportions between the two randomised treatment arms, with a 95% confidence interval. If the upper limit of the 2-sided 95% confidence interval is below 15%, we will declare the intervention arm as non-inferior to the SOC arm.

All binary secondary outcomes will be analysed in the same way described for the primary outcome. The continuous secondary outcomes (duration of neutropenia, total antibiotic duration and total hospital length of stay) will be analysed using a generalised linear mixed model with an identity link function and a normal distribution. This model will be adjusted for baseline covariates and stratification variables. This model will be used to calculate the difference in means between the two randomised treatment arms, with a 95% confidence interval. Variables that are skewed, will be analysed using non-parametric methods (Mann Whitney test) or zero-inflated models, as appropriate.

Participants will be included as a random effect, to consider the fact that the same child can be enrolled more than once. The amount of crossover between treatment arms will be assessed and reported. If the amount of crossover is large, additional statistical methods that can deal with non-randomised comparisons, such as g-methods or inverse probability weighting will be considered.

*Monitoring of adverse events*. Adverse events and adverse reactions (non-serious and serious) will be captured from the time of randomisation for a duration of 28 days. A Serious Adverse Event will include any adverse event/adverse reaction that results in death, is life threatening, requires admission to the ICU, prolongs hospitalisation, results in re-hospitalisation, or results in persistent or significant disability or incapacity. Whilst in hospital, adverse events will be captured daily in the participant EMR and continuously monitored by the study team. If a participant is discharged prior to ANC recovery, the adverse events will be captured on day 28 review of the EMR.

The study investigator will be responsible for determining whether an adverse event is expected or unexpected. An adverse event will be considered unexpected if the nature, severity, or frequency of the event is not consistent with the risk information previously described for the trial intervention. The severity of adverse events will be assessed with reference to the Common Terminology Criteria for Adverse Events (CTCAE) V5.0 produced by the United States National Cancer Institute [34].

Safety oversight will be under the direction of a Clinical Event Committee. The Clinical Event Committee will consist of clinicians and a statistician with respective experience in the management of paediatrics, biostatistics and the conduct and monitoring of randomised controlled trials. Members of the Clinical Event Committee will be independent of the trial study team.

*Safety*. Trial participants and clinicians will not be blinded to trial group assignment to ensure patient safety. Clinicians need to be aware of current and past antibiotic therapy if the participant has a complication such as fever recurrence, bacteraemia or clinical deterioration. Clinicians will have the authority to over-ride the treatment arm, either continue or stop antibiotics, and the justification will be captured. As an additional safety measure, those randomised to the intervention arm will remain in hospital for observation until evidence of ANC recovery. To ensure safety, the research team audited the time to antibiotics for inpatients with onset of FN at the trial site over a 12-month period and found that this met or surpassed international benchmarks.

#### ELSA-Impact methods

In *ELSA-Impact*, quantitative and qualitative data will be collected according to the ‘Theoretical Framework of Acceptability’ (TFA). The TFA consists of seven constructs (affective attitude, burden, ethicality, intervention coherence, opportunity costs, perceived effectiveness, and self-efficacy) and will inform questionnaires administered to clinicians, patients and parents pre-, during- and post-trial recruitment [35, 36].

Exploratory semi-structured focus groups will be conducted with prescribing clinicians (registrar, fellow, junior consultant and senior consultant level) as well as nursing and pharmacy staff caring for children with FN. These groups will explore attitudes towards short duration empiric antibiotics for FN and perceived barriers and enablers to change in antibiotic-prescribing. Focus group meetings will be held separately with patients, parents and carers who were included in the primary study (*ELSA-FN*) and will explore attitudes to antibiotic use during FN, including factors influencing preference for early or late cessation. Participants will be approached by an investigator or delegated member of the trial team in person (for patients and families admitted to hospital for FN care) or via email (for clinicians caring for oncology patients) and provided with the study-specific PICF. A signed PICF must be returned prior to participating in a focus group. Participants are able to withdraw before or after the focus group commences. Participants will be recruited until data saturation is achieved, with an expected sample size of 5–10 people across 4–5 focus groups [37]. The content of the focus groups will be coded by theme and analysed according to the TFA constructs [36]. The EMR meta-data will be harnessed to provide a quantitative measure of acceptability. This will include the proportion of patients who decline participation, deviation from trial protocol and time to randomisation. The within-hospital costs assigned to each FN episode, separated into individual cost buckets, will be compared for the two approaches to FN treatment [13]. This information is readily accessible from hospital information systems. Cost-effectiveness of the intervention will be presented as a cost-minimisation analysis to show the relative impact on costs of stopping antibiotics (intervention), compared with the SOC arm.

#### ELSA-EMR methods

In *ELSA-EMR*, quantitative EMR metadata and qualitative data on the EMR-embedded trial design will be collected according to the Unified Theory of Assessment and Usability of Technology (UTAUT) [38, 39]. The UTAUT has four determinants of intention and usage (performance expectancy, effort expectancy, social influence and facilitating conditions) and has been validated for evaluation of digital health interventions [39, 40]. We will apply the UTAUT questionnaire to establish the relevant domains influencing adoption of EMR-embedded clinical trials. We will conduct focus groups with staff to establish perceived barriers and enablers following the recommendations of the NIH Health Care Systems Research [41]. A signed PICF must be returned prior to participating in any focus group. Participants are able to withdraw before or after the focus group commences.

The costs of the embedded trial will be measured to allow cost-effectiveness analysis and comparison with the costs of traditional trials methodology [26]. The expected costs of this trial design include: (i) labour and software resources to develop, build and embed *ELSA-FN*; (ii) labour and software resources to test, monitor and optimise *ELSA-FN* trial integration with the EMR; and iii) handling and analysis of outcome data extracted from the EMR. Itemised costing estimates will be developed to include the standard rates for key trial personnel. These will be compared to the estimated costs, obtained from the Independent Health and Aged Care Pricing Authority (IHACPA), of running the *ELSA-FN* trial using conventional manual methodology.

*Confidentiality*. All *ELSA-FN* and *ELSA-Impact* participant data will remain confidential. Deidentified *ELSA-FN* datasets created for analysis will contain a unique identifier contact serial number (CSN). Only personnel with EMR access and the ability to search by CSN will be able to identify these patients.

*ELSA-Impact* focus groups will be digitally recorded. Recording and transcription will be deleted at the conclusion of the research project and after a minimum of five years. Participants will be de-identified during hand transcription by the researcher and in any subsequent analysis or publication. Where a participant withdraws consent after the interview has been recorded, their comments will be excluded from hand transcription and analysis.

## Discussion

The *ELSA-FN* trial will address the safety of stopping antibiotics before ANC recovery in children with cancer and high-risk FN. This is identified as a critical research gap, and it is anticipated that the results of *ELSA-FN* will lead to a paradigm shift in FN management internationally. If identified as non-inferior to current standard of care, up to 70% of children with cancer and high-risk FN will avoid prolonged antibiotics and associated known negative impacts on quality of life, antimicrobial resistance, hospital length of stay and healthcare cost.

This patient-centred trial has had active consumer engagement across all aspects, from trial co-design through to assessment of acceptability of the intervention. *ELSA-Impact* will help understand clinician- and patient-factors influencing antibiotic prescribing in FN and directly compare the costs of stopping antibiotics before ANC recovery with current clinical practice. This data will influence design of care pathways for children with FN in the future.

To our knowledge, this is the first Australian paediatric RCT embedded in an EMR and as such, there has been significant investment to design and test the required digital phenotypes that are the foundation of the trial. This has required multidisciplinary collaboration and careful testing to ensure reliability of recruitment and randomisation, as well as close monitoring of data quality. It is hoped that this experience can inform future embedded clinical trials through open-access publication of the digital phenotypes and trial protocol. While embedded clinical trials hold promise to be cost-effective through simplified study infrastructure and incorporation of study activities within routine clinical practice, the true economics have not been fully evaluated. The costs of developing, embedding and running *ELSA-FN* are being measured from the outset to allow a more complete economic analysis and comparison with traditional trials methodology. If shown to be practical and cost-effective, embedded clinical trials could be key to integrating research into the paediatric healthcare environment and improving outcomes for our patients.

## Supporting information

S1 Checklist SPIRIT checklist. (PDF)

S1 File ELSA-FN study protocol version 6.2. (PDF)

S1 Table Clinical definitions and translation to the electronic medical record (S1 Table). (DOCX)
