# Supplementary material for: Protocol for an embedded randomised controlled trial of Early versus Late Stopping of Antibiotics in children with Febrile Neutropenia (ELSA-FN)
Source: PLoS One. 2024 Dec 9;19(12):e0311523. doi: 10.1371/journal.pone.0311523 (PMC11627426; doi:10.1371/journal.pone.0311523)
Supplement: S1 File — (PDF) [file pone.0311523.s002.pdf]

Early versus Late Stopping of Antibiotics in Children with Cancer and High-risk Febrile Neutropenia (ELSA FN)

Protocol Version 6.2 and 29th August 2023
ERM No. 74690

Document history:

Version Number and Date	Summary of changes
1.0	Initial submission
2.0 31/8/21	Updated as detailed in ethics submission
3.0 21/9/21	Minor update to section 11.3 as per ethics response.
4.0 2/12/21	Update to inclusion criteria by adding TOT17 protocol and HSCT out to 100 days post-transplant
5.0 1/9/22	Update to include video as part of consent process
6.0 31/3/23	Addition of qualitative research methods and trial advertising
6.1 25/7/23	Addition of ELSA-FN Impact and detail of focus groups.

CONFIDENTIAL
This document is confidential and is the property of A/Prof Gabrielle Haeusler, Murdoch Children's Research Institute. No part of it may be transmitted, reproduced, published, or used without prior written authorisation from the institution.

Statement of Compliance

This clinical trial will be conducted in compliance with all stipulation of this protocol, the conditions of the ethics committee approval, the NHMRC National Statement on ethical Conduct in Human Research (2007 and all updates), the Integrated Addendum to ICH E6 (R1): Guideline for Good Clinical Practice E6 (R2), dated 9 November 2016 annotated with TGA comments and the NHMRC guidance Safety monitoring and reporting in clinical trials involving therapeutic goods (EH59, 2016).

CONTENTS

PROTOCOL SYNOPSIS 6

GLOSSARY OF ABBREVIATIONS 11

INVESTIGATOR AGREEMENT 13

1. ADMINISTRATIVE INFORMATION 14

1.1. Trial registration 14

1.1.1. Trial registry 14

1.2. Sponsor 14

1.3. Expected duration of study 14

1.4. Contributorship 14

1.5. Stakeholder involvement 15

2. INTRODUCTION AND BACKGROUND 15

2.1. Trial rationale and aim 16

2.2. Background 16

2.3. Risk/Benefit assessment 19

2.3.1. Known potential risks 19

2.3.2. Known potential benefits 20

2.3.3. Assessment of potential risks and benefits 21

3 TRIAL OBJECTIVES AND OUTCOMES 21

3.1 Objectives 21

3.1.1 Primary objective 21

3.1.2 Secondary objectives 21

3.1.3 Exploratory objectives 22

3.2 Outcomes 22

4 TRIAL DESIGN 24

4.1 Overall design 24

4.2 Trial population 24

4.3 Eligibility criteria 24

4.3.1 Inclusion criteria 25

4.3.2 Exclusion criteria 25

4.4 Lifestyle considerations 25
4.5 Screen failures 25
4.6 Recruitment and identification of potential participants 26
4.7 Consent 27
5 INTERVENTION 28
5.1 Intervention arms 28
5.2 Intervention(s) 29
5.2.1 Measurement of participant compliance 29
5.2.2 Other 29
5.2.2.1 Excluded medications and treatments 29
5.2.2.2 Concomitant therapy 29
5.2.3 Discontinuation from trial intervention 29
6 RANDOMISATION AND BLINDING 29
Study participants will be randomly assigned, in a 1:1 ratio into the 2 study groups. A statistician not directly involved in the analysis of the trial results will prepare the randomisation schedule using block randomisation to maintain balance between treatment arms. 29
6.1 Concealment mechanism 29
6.2 Breaking of the trial blind 29
7 TRIAL VISITS AND PROCEDURES 29
7.1 Trial timeline 29
7.2 Schedule of assessments 30
7.3 Description of procedures 30
7.4 Notes on specific trial visits 31
7.4.1 Screening 31
7.4.2 Final trial visit 31
7.5 Treatment discontinuation, participant withdrawals and losses to follow up 31
7.5.1 Discontinuation of treatment - participant remains in trial for follow up 31
7.5.2 Withdrawal of consent - participant withdraws from all trial participation 32
7.5.3 Losses to follow-up 32
7.5.4 Replacements 32
7.5.5 Trial Closure 32
8 SAFETY MONITORING AND REPORTING 33
8.1 Definitions 33
8.2 Capturing and eliciting adverse event/reaction information 34

8.3 Documentation of AEs......34
8.4 Assessing the seriousness of a participant's AE......34
8.5 Assessing the relatedness (causality) of a participant's AE......35
8.6 Assessing the expectedness of a participant's AE......35
8.7 Reporting of safety events......35
9 DATA AND INFORMATION MANAGEMENT......37
9.1 Overview......37
9.2 Data management......37
9.2.1 Data generation (source data)......37
9.2.2 Data capture methods and data use, storage, access and disclosure during the trial 37
9.2.3 Data confidentiality......38
9.2.4 Quality assurance......39
9.2.5 Archiving - Data and document retention......39
9.2.6 Data sharing......40
10 TRIAL OVERSIGHT......40
10.1 Governance structure......40
10.1.1 Trial Management Group (TMG)......40
10.1.2 Trial Steering Committee (TSC)......40
10.1.3 Safety Monitoring......41
10.2 Quality Control and Quality Assurance......41
11 STATISTICAL METHODS......41
11.1 Sample Size Estimation......41
11.2 Population to be analysed......41
11.2.1 Handling of missing data......42
11.3 Methods of analysis......42
11.4 Interim Analyses......43
12 ETHICS AND DISSEMINATION......43
12.1 Research Ethics Approval & Local Governance Authorisation......43
12.2 Amendments to the protocol......43
12.3 Protocol Deviations and Serious Breaches......43
13 CONFIDENTIALITY......44
14 PARTICIPANT REIMBURSEMENT......44
15 FINANCIAL DISCLOSURE AND CONFLICTS OF INTEREST......44

16 DISSEMINATION AND TRANSLATION PLAN 44
17 ADDITIONAL CONSIDERATIONS 44
18 REFERENCES 44
19 APPENDICES 47
19.1 Appendix 1: Division of sponsor responsibilities between sponsor and sponsor-investigator 47
19.2 APPENDIX 2: Significant Safety Issues (SSI) - some examples 49
19.3 APPENDIX 3: Expedited Safety Report Form 50
19.4 APPENDIX 4: eMR Definitions 52
19.5 APPENDIX 5: Antibiotic Prescribing Focus Groups 57
19.6 APPENDIX 6: ELSA-FN Trial video information script 58
19.7 APPENDIX 7: Trial poster 59
19.8 APPENDIX 8: Specimens for biobanking - completed biobank registration form 60

PROTOCOL SYNOPSIS

TITLE	Early versus Late Stopping of Antibiotics in Febrile Neutropenia (ELSA-FN)
TRIAL DESCRIPTION	This randomised controlled non-inferiority trial will compare stopping empiric antibiotics prior to absolute neutrophil count (ANC) recovery (STOP) to standard of care (SOC) in children with cancer and high-risk FN.
OBJECTIVES	***Primary Objective:***<br>1. To determine if stopping antibiotics prior to ANC recovery in children with cancer and high-risk FN is non-inferior to standard of care.<br><br>***Secondary objectives:***<br>To determine the impact that stopping antibiotics prior to ANC recovery, compared with standard of care, has on:<br>1. Recurrence and duration of temperature ≥38 degrees Celsius (ie. new fever episode after afebrile period of ≥48h)<br>2. Clinical instability (one or more of conscious state, respiratory rate, blood pressure, oxygen saturation meeting mandatory emergency call criteria OR two or more respiratory rate, blood pressure, heart rate or oxygen saturations simultaneously [+/-4h] meeting clinical review criteria)<br>3. Admission to intensive care unit for organ support<br>4. New positive blood culture<br>5. 28 day all-cause and infection-related mortality<br>6. Duration of neutropenia (measured as days from initial ANC <500 cells/mm^3 to ANC ≥500 cells/mm^3)<br>7. Total antibiotic duration (days of therapy)<br>8. Total hospital length of stay (LOS)<br>9. Readmission to hospital (inpatient ward) within 28 days of randomisation<br>10. Development of *Clostridioides difficile* infection<br>11. Development of an antibiotic resistant infection or colonisation within 28 days of randomisation<br><br>ELSA-Impact:<br>12. Clinician confidence and acceptability<br>13. Patient/parent/caregiver confidence and acceptability<br>14. Cost effectiveness

OUTCOMES AND OUTCOME MEASURES	***Primary Outcome***<br>**Unfavourable clinical course** defined as any of the following occurring after randomisation and during the same period of severe neutropenia (ANC <500 cells/mm^3):<br>• Recurrence of temperature ≥38 degrees Celsius (ie. new fever episode after an afebrile period of at least 48h);<br>• Clinical instability (one or more of conscious state, respiratory rate, blood pressure, heart rate, oxygen saturation meeting mandatory emergency call criteria OR two or more respiratory rate, blood pressure, heart rate or oxygen saturations simultaneously (+/- 4h) meeting clinical review criteria)<br>• Admission to the intensive care unit<br>• New positive blood culture collected after randomisation (with any organism)<br>• Death<br><br>***Secondary outcomes***<br>Defined as any of the following <u>within 28 days of randomisation</u> (unless otherwise stated):<br>• Fever (temperature ≥38 degrees Celsius):<br>o Recurrence of fever during the same period of neutropenia and/or 28 days of randomisation<br>o Duration (hours) of temperature ≥38 degrees Celsius during the same period of neutropenia and/or 28 days of randomisation<br>• Clinical instability (one or more of conscious state, respiratory rate, blood pressure, heart rate, oxygen saturation meeting mandatory emergency call criteria OR two or more respiratory rate, blood pressure, heart rate or oxygen saturations simultaneously (+/- 4h) meeting clinical review criteria) during the same period of neutropenia and/or 28 days of randomisation<br>• Admission to intensive care unit during the same period of neutropenia and/or 28 days of randomisation<br>• Admission to the intensive care unit for organ support during the same period of neutropenia and/or 28 days of randomisation<br>o Vasopressor/inotrope therapy<br>o Renal replacement therapy<br>o Invasive or non-invasive ventilation<br>• New positive blood culture during the same period of neutropenia<br>• New infection after randomisation and during the same period of neutropenia and/or 28 days of randomisation, including:

	o Microbiologically defined infection (MDI): infection that is clinically documented and microbiologically confirmed<br>o Clinical documented infection (CDI): an infection that is clinically detectable but no pathogen is identified<br>o Fever without focus: Fever without a documented MDI or CDI<br>• Mortality:<br>o All cause 28-day mortality<br>o Infection-related 28-day mortality (death with microbiologically proven or clinically suspected infection)<br>• Duration of neutropenia (measured as days from ANC <500 cells/mm^3 to ANC ≥500 cells/mm^3)<br>• Antibiotic duration<br>o Total antibiotic duration measured as length of therapy (LOT) from randomisation and within 28 days of randomisation (excluding antibiotic prophylaxis)<br>o Total antibiotic duration measured as days of therapy (DOT) from randomisation and within 28 days of randomisation (excluding antibiotic prophylaxis)<br>o Re-instalment of antibiotic (STOP arm) – yes/no<br>• Total hospital length of stay (LOS) in days from randomisation to hospital discharge<br>• Unplanned readmission to hospital (inpatient ward) within 28 days of randomisation<br>• *C. difficile* infection within 28 days of randomisation<br>• Antibiotic resistant (MRSA, ESBL-producing enterobacterales, CRE, VRE) infection or colonisation within 28 days of randomisation<br><br>ELSA-Impact:<br>• Clinician confidence and acceptability:<br>o Number of patients for which randomisation is overridden in STOP arm<br>o Reason for continuing antibiotics in STOP arm: i) Presence of patient-specific risk factors; ii) Presence of clinically-defined infection; iii) Clinical preference of treating oncologist; iv) Other reason<br>o Clinician discussion of antibiotic prescribing process<br>• Patient/parent/carer confidence and acceptability:<br>o Number of patients that consent to the study as proportion of patients eligible.<br>o Number of patients for which randomisation is overridden in STOP arm due to withdrawn consent

	○ Patient and parent/carer discussion of antibiotics in FN<br>• Within-hospital costs assigned to each FN episode and separated into individual cost buckets
TRIAL POPULATION	*Inclusion criteria:*<br>• Children (age <18y) with any of acute myeloid leukaemia (AML); acute lymphoblastic leukaemia (ALL) in dose intensive phases of induction/re-induction, intensification and consolidation; ALL or acute lymphoblastic lymphoma on TOT17 protocol; or within 100 days of allogeneic or autologous HSCT ; AND<br>• Neutropenia (<500 cells/mm^3); AND<br>• Commenced on empiric FN antibiotics (any of: piperacillin-tazobactam, cefepime, ceftazidime, ciprofloxacin, +/- vancomycin, +/-amikacin); AND<br>• Afebrile (temperature <38.0°C) period for at least 48 hours and no more than 96 hours after at least one temperature measured by axillary or tympanic thermometer (≥38.0°C); AND<br>• Clinical stability for at least 48 hours (no conscious state, respiratory rate, blood pressure, or oxygen saturations in mandatory MET call criteria OR heart rate in the clinical review criteria [> 95th percentile for age] in 48 hours prior to randomisation)<br><br>*Exclusion criteria:*<br>• Prolonged febrile neutropenia (documented daily temperature ≥38.0°C for ≥ 5 days)<br>• Documented positive blood culture since onset of FN episode and prior to randomisation (microbiologically defined infection)<br>• Documented other infection requiring antibiotic treatment (microbiologically or clinically defined infection)<br>• Admitted to ICU at time of randomisation<br>• Within 28 days of last randomisation<br><br>Target sample size 312 participants (156 in each arm)<br><br>See Appendix 5 for trial population and sample size for Secondary Objectives 12-14 (ELSA-Impact)
DESCRIPTION OF SITES ENROLLING PARTICIPANTS	Single Site – Royal Children's Hospital, Melbourne

DESCRIPTION OF INTERVENTIONS	*Intervention:* Stopping empiric FN antibiotics after resolution of fever for at least 48 hours, irrespective of ANC (STOP)<br>*Control*: Continuing empiric FN antibiotics until resolution of fever for at least 48 hours and recovery of ANC to greater than 500 cells/mm^3 (SOC)
TRIAL DURATION	48 months
PARTICIPANT DURATION	30 days

GLOSSARY OF ABBREVIATIONS

ABBREVIATION	TERM
AE	adverse event
ALL	acute lymphoblastic leukaemia
AML	acute myeloid leukaemia
AMR	antimicrobial resistance
ANC	absolute neutrophil count
BSI	blood stream infection
CAPA	corrective and preventative action plan
CDI	clinically defined infection
CHA	chief health analyst
CPG	clinical practice guideline
CRE	carbapenem-resistant Enterobacteriaceae
CRF	case report form
CTAE	common terminology criteria for adverse events
DOT	days of therapy
DSMB	data and safety monitoring board
ECIL	European conference on infections in leukemia
eMR	electronic medical record
ESBL	extended spectrum beta-lactamase
FN	febrile neutropenia
G-CSF	granulocyte colony stimulating factor
HITH	hospital in the home
HREC	human research ethics committee
HSCT	haematopoetic stem cell transplant
ICU	intensive care unit
JMML	juvenile myelomonocytic leukaemia
LOS	length of stay
LOT	length of therapy
MDI	microbiologically defined infection
MET	medical emergency team
MRSA	methicillin resistant Staphylococcus aureus
NHMRC	national health and medical research council
PI	primary investigator
QC	quality control
QOL	quality of life
RAE	related adverse event
RCH	Royal Children's Hospital (Melbourne)
RCT	randomised controlled trial
RGO	research governance officer
SAE	serious adverse event
SOC	standard of care
SOPs	standard operating procedures

SSI	significant safety issue
TMG	trial management group
TMP-SMX	trimethoprim-sulfamethoxazole
TOT17	Total Therapy Study 17 for Newly Diagnosed Patients with Acute Lymphoblastic Leukemia and Lymphoma
TSC	trial steering committee
TTA	time to antibiotics
URSAE	unexpected and related serious adverse event
USM	urgent safety measure
VRE	vancomycin-resistant Enterococci

INVESTIGATOR AGREEMENT

I have read the protocol entitled Early versus Late Stopping of Antibiotics in Febrile Neutropenia (ELSA-FN). By signing this protocol, I agree to conduct the clinical trial, after approval by a Human Research Ethics Committee or Institutional Review Board (as appropriate), in accordance with the protocol, the principles of the Declaration of Helsinki and the good clinical practice guidelines adopted by the TGA [Integrated Addendum to ICH E6 (R1): Guideline for Good Clinical Practice E6 (R2), dated 9 November 2016 annotated with TGA comments].

Changes to the protocol will only be implemented after written approval is received from the Human Research Ethics Committee or Institutional Review Board (as appropriate), with the exception of medical emergencies.

I will ensure that trial staff fully understand and follow the protocol and evidence of their training is documented on the trial training log.

Name	Role	Signature and date
A/Prof Gabrielle Haeusler	Sponsor-Investigator	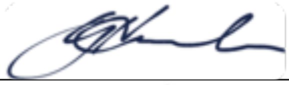
Dr Coen Butters	Co-investigator	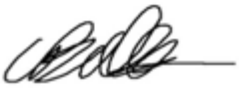

1. ADMINISTRATIVE INFORMATION

1.1. Trial registration

1.1.1. Trial registry

This trial is registered on ClinicalTrials.gov with identifier NCT04948463.

1.2. Sponsor

**Trial Sponsor**	**Murdoch Children's Research Institute**
**Contact name**	Alannah Rudkin
**Address**	**MCRI**
**Sponsor-Investigator** *(where applicable)*	A/Prof Gabrielle Haeusler

On behalf of the Sponsor, MCRI, the Sponsor-Investigator leading the trial will undertake and/or oversee those Sponsor responsibilities delegated by the Sponsor. The delegated Sponsor responsibilities are documented in Appendix 1 of the protocol.

1.3. Expected duration of study

The expected duration of the recruitment period is 36 months. Participants will be followed up for 28 days after the end of their FN episode.

1.4. Contributorship

Name	Summary of contribution
A/Prof Gabrielle Haeusler, MCRI and RCH Infectious Diseases	Study conception, design and protocol completion and supervision
Prof Andrew Davidson, MCRI and RCH Clinical Trials	Study conception and design and protocol review and approval
Dr Coen Butters, MCRI	Study design, protocol completion and approval
Prof Jim Buttery Centre for Health Analytics, Melbourne Children's Campus	Study design, protocol review and approval
Prof Karin Thursky, Peter MacCallum Cancer Centre Infectious Diseases	Study design, protocol review and approval

Name	Summary of contribution
Dr Anneke Grobler, Murdoch Children's Research Institute	Protocol design, review and approval
Alannah Rudkin, RCH Children's Cancer Centre	Protocol design, review and approval
Dr Theresa Cole, RCH Children's Cancer Centre	Protocol design, review and approval
Dr Diane Hanna, RCH Children's Cancer Centre	Protocol design, review and approval
Dr Stacie Wang, RCH Children's Cancer Centre	Protocol design, review and approval
Heather Weerdenburg, RCH Cancer Pharmacy	Protocol design, review and approval
Cindy Bakos, Consumer representative	Protocol design, review and approval

1.5. Stakeholder involvement

Key stakeholders have been involved in the development of this protocol. A consumer representative is part of the project steering group and has been involved in the design of the trial. The protocol was reviewed by three consumer representatives. As outlined in section 1.4 above, the project team includes clinicians involved in the care of children with cancer and high-risk FN (ie. oncology and infectious diseases). The team also includes leading experts in clinical trials and health informatics. The trial is endorsed by the RCH Children's Cancer Centre Clinical Trials Group. Finally, the protocol has been reviewed and endorsed by an international group of clinicians and researchers in the area of infections in children and FN guideline development including Dr Lillian Sung (Oncologist, Hospital for Sick Kids, Toronto), Dr Thomas Lehrnbecher (Oncologist, Johann Wolfgang Goethe-University, Frankfurt), Dr Roland Amman (Oncologist, University of Bern, Bern) and Dr Bob Phillips (Oncologist, University of York, York).

2. INTRODUCTION AND BACKGROUND

Febrile neutropenia (FN) is one of the most common complications of childhood cancer treatment and a leading cause of unplanned or prolonged hospital admission.[1] Management

traditionally involves administration of broad spectrum antibiotics until resolution of fever and recovery of absolute neutrophil count (ANC). However, despite the frequency with which FN occurs there is limited evidence to inform this approach, particularly in high-risk FN. In recognition of the potential harms of prolonged antibiotic exposure, in particular on development of antimicrobial resistance[2,3], optimal antibiotic duration in paediatric FN has been identified as a critical research gap by international experts.[2-4]

2.1. Trial rationale and aim

The overall aim of this study is to determine if stopping empiric antibiotics prior to absolute neutrophil count (ANC) recovery, in children with cancer or following haematopoietic stem cell transplant (HSCT) and febrile neutropenia (FN) is non-inferior to standard of care. This study design was chosen in discussion with key stakeholders from oncology, infectious diseases and consumer representatives. It is based on the assumption that the primary outcome "unfavourable clinical course" occurs with similar frequency in both arms and, the known potential benefits of short course antibiotics will impact acceptability of the results.

2.2. Background

Neutropenia is a predictable consequence of chemotherapy and the severity and duration of neutropenia increases the risk of infection. Children with cancer are heterogenous and should be stratified into low and high-risk based on the statistical risk of infection or adverse outcome.[5,6] Children with cancer considered high-risk of infection or adverse outcome include those with acute myeloid leukemia (AML), acute lymphoblastic leukaemia (ALL) during phases of dose-intensive chemotherapy and post allogeneic haematopoietic stem cell transplant (HSCT) in the pre-engraftment phase. Common across these patient groups is an expected duration of severe neutropenia (defined as ANC <500 cells/mm^3) longer than 7 days. Despite the risk of severe infection, less than 25% have a documented blood stream infection (BSI) and adverse outcome such as ICU admission occurs in less than 3%.[6] While early recognition of fever and administration of antipseudomonal beta-lactam antibiotics (eg. piperacillin-tazobactam or cefepime) is recommended in all national and international guidelines, recommendations for antibiotic duration are conflicting (Table 1).

Table 1. Antibiotic duration in febrile neutropenia as recommended by current international guidelines		
**Guideline**	**Publication year**	**Recommended duration of empiric antibiotics in FN**
Evidence-Based Recommendations for Antimicrobial Use in Febrile Neutropenia in Japan (Masaoka T *et al*)	2004	Low-risk: continue until a minimum of 15 days without fever<br>High-risk: continue until neutrophil recovery
Infectious Diseases Society of America (IDSA)	2010	Continue until patient is afebrile and ANC >500 cells/mm^3
Eighth European Conference on Infections in Leukemia (ECIL-8)	2020	Low-risk: continue for ≥ 72 hrs and until afebrile for ≥ 48 hours, regardless of ANC or expected duration of neutropenia
European Society for Medical Oncology (ESMO)	2016	Low-risk: Continue until afebrile for 5-7 days (or ANC > 500 cells/mm^3)<br>High-risk: Continue for up to 10 days (or until ANC > 500 cells/mm^3)
International Pediatric Fever and Neutropenia Guideline (Lehrnbecher T *et al*)	2017	Continue until resolution of fever for 24 hours and evidence of bone marrow recovery
UK National Institute of Health and Care Excellence (NICE)	2020	Continue until "response to treatment" (irrespective of neutrophil count)

The 2017 International Pediatric Fever and Neutropenia Guideline recommends "continuing empiric antibiotics in high-risk patients until resolution of fever for 24 hours and evidence of bone marrow recovery", however the authors note that the "optimal duration of empirical antibiotics for high-risk patients with sustained bone marrow suppression was not addressed in the systematic review and continues to be an important research gap."[5] The Infectious Diseases Society of America (IDSA) guidelines similarly recommends continuing empirical antibiotics until the patient is afebrile and ANC >500 cells/mm^3, based on just two studies demonstrating a risk of recurrent fever and sepsis in adults with persisting neutropenia.[7] The European Society for Medical Oncology recommends continuing empiric antibiotics for up to 10 days or until ANC >500 cells/mm^3.[8] In contrast, the recent National Institute of Health and Care Excellence (NICE) guideline recommends discontinuing empiric antibiotic therapy in patients whose neutropenic sepsis has responded to treatment, irrespective of neutrophil count.[9] The Fourth European Conference on Infections in Leukemia (ECIL-4) guidelines, state that empirical antibiotics may be stopped after 72 hours or more in people who have been afebrile for 48 hours and are stable, irrespective of neutrophil count or expected duration of neutropenia.[10]

There are just eight RCTs in FN that compare prolonged (ie. continuation of antibiotics until recovery of ANC) with short course (ie. discontinuation of antibiotics irrespective of ANC) antibiotic therapy in FN.[11] Only two have been conducted since the year 2000 and only one study focused on the high-risk patient population (AML, ALL and HSCT) and excluded children.[12] Despite these limitations, short course antibiotics appear to reduce antibiotic exposure without an increase in adverse event or bacterial infection.[11] Of the three

paediatric-specific RCTs published to date, only two included higher-risk FN episodes (Table 2).[13,14] In the first trial (n=75) which included 42% higher-risk FN episodes, stopping antibiotics at 72 hours in clinically stable children with CRP ≤ 40 mg/L was not associated with higher rates of clinical failure.[13] In the second study (n=176) which included 50% (n=88) higher-risk FN episodes, stopping antibiotics at 48hrs in children with confirmed upper respiratory tract infection, resulted in uneventful resolution of FN in >96% in both arms.[14] While observational FN data is in keeping with these results, the paucity of paediatric RCT-level data limit the strength of recommendations for antibiotic duration in international paediatric FN guidelines and is a key barrier to optimising care in these patients.[5]

Table 2. Randomised prospective studies of short course antibiotics in children with cancer and febrile neutropenia

Study	Number	Inclusion/Exclusion criteria	Randomisation Intervention	Outcome	Short course arm	Results
**Santolaya 1997**[13]<br><br>Placebo RCT<br>Single centre<br>Chile<br>1994-1996	N=75<br>-Short: 36<br>-Long: 39	**Inclusion**<br>-Children with FN<br>-PUO<br><br>**Exclusion**<br>-CDI or MDI (bacterial)<br>-Serum CRP≥4 on day1 or 2<br>-Haemodynamic instability<br>-Positive admission cultures	Day 3 (48h) after admission<br><br>Intervention (Group A): stop D3<br><br>Comparator (Group B): continue until fever and neutropenia resolved	Clinical failure (any of): focus suggestive of a bacterial infection, a positive bacterial culture > D3, fever recurrence, haemodynamic instability, or increase in serum CRP > 40 in 2 consecutive measurements	**Demographic:**<br>- Leuk 15 (42%)<br>- Induction 27 (75%)<br><br>**Outcome:**<br>- 2 (5.6%)<br><br>**Failures/events:**<br>Deaths: 0<br>Bacterial infection: 2<br>PICU:0<br>Sepsis:0	**No difference in:**<br>-LOS<br>-Adverse event
**Santolaya 2017**[14]<br><br>RCT (open)<br>Multisite(n=5)<br>Chile<br>2012-2015	N=176<br>-Short: 84<br>-Long: 92	**Inclusion**<br>-Children with FN<br>-NPA positive for virus<br><br>**Exclusion**<br>-HSCT<br>-Any documented bacterial infection<br>-Any clinical foci of infection<br>-Unfavourable evolution before randomisation (fever, CRP ≥ 90 mg/L, haemodynamic instability)	Day 3 (48h) after admission<br><br>Intervention (Group A): stop D3<br><br>Comparator (Group B): Stop D7 days if afebrile for 24 hours and CRP < 40 mg/L	Uneventful resolution	**Demographic**<br>-Leuk 55 (66%)<br>-HR 45 (54%)<br><br>**Outcome**<br>-80 (95.2%)<br><br>**Events (any of):**<br>-Failure: 4 (4.8)<br>-Restart abs:4<br>-Deaths:0<br>-Bact infection:1<br>-PICU:0<br>-Sepsis:0	**Significant reduction**<br>-Ab duration (median 3 v 7d)<br><br>**No difference in:**<br>-LOS: 6v6d<br>-Adverse event: 5%v3%<br>-Bact infection: 1%v2%<br>-Sepsis, PICU, death:0

In the absence of safety and efficacy data for short course antibiotics, the median duration of broad-spectrum antibiotics in children at RCH with high-risk FN who do not have an identified infection, prolonged fever or adverse outcome, is 6.3 days (IQR 3.5-13.5d) (Table 3). Furthermore, almost 30% of these patients experience new fever episodes or breakthrough bacteraemia, despite continuing antibiotics, prompting medical review and adjustments of therapy (Table 3). Taking this into consideration, early cessation of antibiotics may translate to reduced antibiotic exposure in up to 70% of patients.

This is the first study of its kind in children with high-risk FN and will address critical research gaps and variation in clinical practice. Results will influence both national and international paediatric FN guidelines and, given the pragmatic trial design, could be rolled out to Parkville precinct Epic partners.

Table 3. Baseline data from PICNICC national FN study
NB. HSCT data from local RCH study

	All AML	AML (excl BSI or ICU in 1st 48h)	ALL**	ALL** (excl BSI or ICU in 1st 48h)	All lymphoma induction	Lymphoma induction (excl BSI or ICU in 1st 48h)	All HSCT^	TOTAL (excl BSI or ICU in 1st 48h)
**Numbers episodes at RCH over 12m [patients]**	18	14	81	64	4	3	38 [?]	81^
**Total across Australian PICNICC study**	**67**	**52**	**264**	**215**	**13**	**12**	**NA**	**279**
**Inpatient onset, n (%)**	46 (68.7)	36 (69.2)	208 (78.8)	40 (18.6)	3 (23.1)	2 (16.7)	(100)	78 (28.0)
**Primary true BSI, n (%)**	16 (23.9)	4 (7.7)	48 (18.2)	4 (1.9)	1 (7.7)	0	Pending	8 (2.9)
**2nd BSI >48h**	6 (8.9)	6 (11.5)	16 (6.1)	11 (5.1)	0	0	NA	17 (6.1)
**Median duration primary fever, d (IQR)**	1.1 (0.3-3.6)	1.0 (0.2-2.4)	0.5 (0.1-2.1)	0.5 (0.03-1.9)	1.1 (0.1-3.7)	0.7 (0.1-3.4)	Pending	0.6 (0.1-2.0)
**2nd fever**	30 (44.8)	24 (46.2)	67 (25.4)	54 (25.1)	3 (23.1)	2 (16.7)	30%	80 (28.7)
**ICU admission (any)**	4 (6.0)	1 (1.9)	13 (4.9)	4 (1.9)	0	0	Pending	5 (1.8)
**ICU admission >48h, n (%)**	2 (3.0)	1 (1.9)	5 (1.9)	4 (1.9)	0	0	Pending	5 (1.8)
**Median Ab duration, d (IQR)**	13.8 (7.8-20.8)	12.6 (6.8-19.3)	6.7 (3.7-13.6)	5.5 (3.2-11.4)	4.7 (3.0-7.6)	5.8 (4.2-7.5)	Pending	6.3 (3.5-13.5)
**Hospital LOS, days (IQR)**	15.7 (9.9-27.7)	14.8 (9.4-27.4)	6.9 (3.9-13.9)	6.3 (3.6-12.7)	5.2 (3.0-14.8)	4.9 (2.8-11.2)	Pending	7.1 (3.7-14.8)
**Severe neutropenia duration, days (IQR)***	16.1 (9.6-21.1)	16.2 (9.6-21.5)	10.8 (5.5-17.5)	11.1 (5.6-17.6)	6.0 (4.2-7.6)	5.8 (4.2-7.5)	Pending	11.1 (5.6-17.7)
**Mortality – 30d all cause**	1 (1.5)	1 (1.9)	2 (0.8)	1 (0.5)	0	0	Pending	2 (0.7)
**Composite outcome***	**32 (47.8)**	**25 (48.1)**	**73 (27.7)**	**56 (26.0)**	**3 (23.1)**	**2 (16.7)**	Pending	**83 (29.7)**

* calculated from FN presentation or inpatient onset of FN until ANC >0.5
**ALL in induction, consolidation, DI or other intensive non-COG phase
^excluding HSCT data as preliminary data pending – based on 80% eligibility rate (279/344 = 81%), 30 patients with HSCT eligible for study making total eligible at RCH 111 per year.

2.3. Risk/Benefit assessment

2.3.1. Known potential risks

The potential immediate risks of early cessation of antibiotics in children with high-risk FN are new or missed bacteraemia and physiological instability such as sepsis and/or need for ICU admission. While death due to FN is reported, this is an exceedingly rare event in children in developed healthcare settings (0 infection-related deaths in a prospective cohort of 858 FN episodes in Australia).[6] Deaths in FN are mostly attributable to delayed recognition and treatment, including antibiotics administered >60 minutes.[15] Historical experience of perceived poor outcomes such as break through bacteraemia and clinical instability in FN likely informs the current clinical practice of continuing antibiotics until resolution of neutropenia, despite paucity of RCT-level data to guide this approach. Furthermore, prospectively collected local data indicate that these events can occur in up to 30% of patients with high-risk FN who remain on broad-spectrum antibiotics despite the absence of a documented infection or clinical instability in the first 48 hours (Table 2). In addition to this, in the eight RCTs that have investigated early cessation of antibiotics in FN, including a similar high-risk adult FN population, there was no increased risk of serious adverse events in the intervention arm compared to controls.[11,12]

To mitigate these potential risks, all patients enrolled in the trial will remain in hospital at RCH until resolution of severe neutropenia. They will be placed under "neutropenic observation" with antibiotics pre-prescribed on an existing high-risk FN care pathway as per current standard practice at RCH. This pathway was implemented in 2018 to avoid unnecessary

antibiotic delays for high-risk patients and enables pre-prescribed antibiotics to be nurse initiated in patients meeting specified criteria (clinical instability or temperature ≥38.0°C). Using this pathway, the median time to first dose antibiotics at RCH is 22mins (range 5-40 mins) and median time to medical review is 40 mins (range 0-220 mins).[16]

Randomisation will occur at least 48 hours after the last documented fever. Australian prospective FN data indicate that 95% of all clinically significant blood stream infections are identified within the first 48 hours.[17] In the unlikely event that a patient is randomised to cease antibiotics at exactly 48 hours after the first blood culture is taken (ie. median duration of primary fever is 14 hours so 50% patients randomised at 62 hours), they will remain in hospital and antibiotics can be re-commenced promptly on notification of blood culture result or if any clinical concern.

We do not anticipate any social, legal or economic risks to the patients enrolled in this trial. While there may be psychological stress for families enrolled in the short course antibiotic arm due to concerns about the potential for an adverse event, we anticipate this will be mitigated by the treating teams routine clinical review and remaining in hospital under close observation. Furthermore, children undergoing reduced intensity therapy for FN (ie. home-based or oral antibiotics) may have improved quality of life.[18,19]

We do not anticipate any long-range risks of early cessation of antibiotics. The intervention in this study will occur within the discrete FN episode. It is anticipated to reduce antibiotics duration by approximately 4 days (Table 2).[14] Following resolution of fever and ANC recovery (ANC ≥500 cells/mm^3) the FN episode is completed and antibiotics are usually ceased and patients will enter their next cycle of chemotherapy.

2.3.2. Known potential benefits

Published RCTs have shown a reduction in antibiotic days and length of hospital stay in children with FN receiving short course antibiotics.[11] Children classified as high-risk of FN have high rates of antibiotic exposure and the current standard of care, continuation of antibiotics until resolution of neutropenia, adds significant antibiotic treatment days over the course of cancer treatment. Antibiotic exposure in the paediatric cancer population is associated with a range of potential harms. Antibiotics in children, while mostly well tolerated, have a range of immediate adverse effects such as nausea, vomiting, nephrotoxicity, electrolyte disturbances, hypersensitivity reactions and added myelosuppression.[20] Prior and prolonged antibiotic exposure is also a risk factor for infection with antimicrobial resistant (AMR) bacteria[2,21] and those that develop these infections have a poorer outcome, including an increased risk of death.[2]

In addition to increased risk of AMR, antibiotic exposure is also associated with increased rates of invasive candidiasis and infection with *Clostridioides difficile.*[22,23] Recent studies have also shown an association between antibiotic exposure and graft-versus-host disease in patients undergoing HSCT, potentially through modification of gut microbiota.[24] The interaction between antibiotics and chemotherapy agents is not well understood. However, for immune

checkpoint inhibitors, a targeted cancer immunotherapy, prior antibiotic use is associated with worse treatment response and decreased overall survival.[25,26]

2.3.3. Assessment of potential risks and benefits

Increasing worldwide antibacterial resistance represents a global health risk and has significant implications for the treatment of infections in children with cancer. Research should drive efforts to optimise use of antibiotics and safely minimise duration of therapy in this population. Current international guidelines provide conflicting recommendations on the duration of antibiotics in high-risk FN and are not informed by robust clinical evidence. By demonstrating the non-inferiority of short course antibiotics there is the potential to improve both the quality and cost of supportive care for children with cancer worldwide. This is balanced against the low but potential risk of physiological instability or recurrent fever in children in the intervention arm.

Based on local data, bacteraemia in children with high-risk FN is uncommon and almost entirely occurs within the first 48 hours of treatment. As patients randomised to short course antibiotic therapy will remain as admitted patients they will continue to receive routine monitoring. RCH has an established management pathway for children with febrile neutropenia including proven timely access to medical review and initiation, modification or re-commencement of antibiotics.

3 TRIAL OBJECTIVES AND OUTCOMES

3.1 Objectives

3.1.1 Primary objective

To determine if stopping antibiotics prior to ANC recovery (ANC ≥ 500 cells/mm^3) in children with cancer and high-risk FN is non-inferior to standard of care.

3.1.2 Secondary objectives

The ***secondary objectives*** of this study are to determine the impact that stopping antibiotics prior to ANC recovery, as compared to standard of care, has on:

1. Recurrence of temperature ≥38 degrees Celsius (ie. new fever episode after afebrile period of 48h)
2. Clinical instability (one or more of conscious state, respiratory rate, blood pressure, heart rate, oxygen saturation meeting mandatory emergency call criteria OR two or more respiratory rate, blood pressure, heart rate or oxygen saturations simultaneously (+/- 4h) meeting clinical review criteria)
3. Admission to intensive care unit
4. Admission to intensive care unit for organ support
5. New positive blood culture
6. 28 day all-cause and infection-related mortality
7. Duration of neutropenia (measured as days from ANC <500 cells/mm^3 to ANC ≥500 cells/mm^3)

8. Total antibiotic duration (days of therapy)

9. Total hospital length of stay (LOS)
10. Readmission to hospital (inpatient ward) within 28 days of randomisation
11. Development of *C. difficile* infection
12. Development of an antibiotic resistant infection or colonisation within 28 days of randomisation

ELSA-Impact:

13. Clinician confidence and acceptability
14. Patient/parent/caregiver confidence and acceptability
15. Cost effectiveness

3.1.3 Exploratory objectives

Nil

3.2 Outcomes

The **primary outcome** is 'unfavourable clinical course' defined as any of, and occurring during the same period of severe neutropenia (ANC <500 cells/mm^3):

- Recurrence of temperature ≥38 degrees Celsius (ie. new fever episode after afebrile period of at least 48h);
- Clinical instability (one or more of conscious state, respiratory rate, blood pressure, heart rate, oxygen saturation meeting mandatory emergency call criteria OR two or more respiratory rate, blood pressure, heart rate or oxygen saturations simultaneously (+/- 4h) meeting clinical review criteria)
- Admission to the intensive care unit
- New positive blood culture collected after randomisation (with any organism)
- Death

Secondary outcomes

Defined as any of the following (occurring within 28 days of randomisation, unless otherwise stated):

- Fever (temperature ≥38 degrees Celsius):
  - Recurrence of fever during the same period of neutropenia and/or 28 days of randomisation
  - Duration (hours) of temperature ≥38 degrees Celsius during the same period of neutropenia and/or 28 days of randomisation
- Clinical instability (one or more of conscious state, respiratory rate, blood pressure, heart rate, oxygen saturation meeting mandatory emergency call criteria OR two or more respiratory rate, blood pressure, heart rate or oxygen saturations simultaneously (+/- 4h) meeting clinical review criteria) during the same period of neutropenia and/or 28 days of randomisation
- Admission to intensive care unit during the same period of neutropenia and/or 28 days of randomisation
- Admission to the intensive care unit for organ support during the same period of neutropenia and/or 28 days of randomisation:
  - Renal replacement therapy

  - Invasive or non-invasive ventilation
  - Vasopressor/inotrope therapy
- New positive blood culture during the same period of neutropenia
- New infection after randomisation and during the same period of neutropenia and/or 28 days of randomisation, including:
  - Microbiologically defined infection (MDI): infection that is clinically documented and microbiologically confirmed
  - Clinical documented infection (CDI): an infection that is clinically detectable but no pathogen is identified
  - Fever without focus: Fever without a documented MDI or CDI
- Mortality:
  - All cause 28-day mortality
  - Infection-related 28-day mortality (death with microbiologically proven or clinically suspected infection)
- Duration of neutropenia (measured as days from ANC <500 cells/mm^3 to ANC ≥500 cells/mm^3)
- Antibiotic duration
  - Total antibiotic duration measured as length of therapy (LOT) from randomisation and within 28 days of randomisation (excluding antibiotic prophylaxis)
  - Total antibiotic duration measured as days of therapy (DOT) from randomisation and within 28 days of randomisation (excluding antibiotic prophylaxis)
  - Re-instalment of antibiotic (STOP arm) – yes/no
- Total hospital length of stay (LOS) in days from randomisation to hospital discharge
- Unplanned readmission to hospital (inpatient ward) within 28 days of randomisation
- *C. difficile* infection within 28 days of randomisation
- Antibiotic resistant (MRSA, ESBL-producing enterobacterales, CRE, VRE) infection or colonisation within 28 days of randomisation
- Clinician confidence and acceptability:
  - Number of patients for which randomisation is overridden in STOP arm
  - Reason for continuing antibiotics in STOP arm: i) Presence of patient-specific risk factors; ii) Presence of clinically-defined infection; iii) Clinical preference of treating oncologist; iv) Other reason
  - Clinician discussion of antibiotic prescribing process (see 19.5 Appendix 5)
- Patient/parent/carer confidence and acceptability:
  - Number of patients that consent to study as proportion of patients eligible.
  - Number of patients for which randomisation is overridden in STOP arm due to withdrawn consent
  - Patient and parent/carer discussion of antibiotics in FN (see 19.5 Appendix 5)

Exploratory outcomes

Nil

4 TRIAL DESIGN

4.1 Overall design

- **Type of trial**: Embedded, randomised, controlled, non-inferiority trial design – children with high-risk FN will be randomised to early stopping of antibiotics or standard of care. Study inclusion and exclusion criteria, randomisation and outcome data collection will be embedded into the Epic eMR.
- **Intervention**: Stopping empiric FN antibiotics after resolution of fever for 48 hours, irrespective of absolute neutrophil count (ANC)
- **Control**: Continuing empiric FN antibiotics until resolution of fever for 48 hours and recovery of ANC as defined by the treating clinician but usually to ≥ 500/mm^3 ("standard of care, SOC")
- **Number of subjects:** 312 children with high-risk FN with will randomly assigned to short-course antibiotics or standard of care and followed up for 28 days.
- **Non-inferiority margin:** The sample size is calculated with a non-inferiority margin of 15% between the two proportions. While clinical drug trials typically use a small non-inferiority margin, a larger non-inferiority margin of 10-20% is considered acceptable for evaluation of a clinical intervention. The non-inferiority margin of 15% was chosen after discussion with international experts in febrile neutropenia and a cross-sectional group of paediatric oncologists. Given the rarity of primary outcomes such as death, clinical instability or intensive care admission, this non-inferiority margin was chosen with reference to recurrence of fever. As 30% of patients are projected to have recurrence of fever in the SOC arm (see Table 3), it was deemed clinically acceptable for up to 45% of patients in the STOP arm (margin +15%) to have fever recurrence.
- **Blinding:** Non-blinded study with automated collection of outcomes.
- **Site**: Single centre at Royal Children's Hospital, Melbourne
- **Sub-studies**: ELSA-Impact. Factors influencing clinician and patient/parent attitudes towards and acceptance of short course antibiotics and cost-effectiveness of this approach.

4.2 Trial population

The eligible trial population will include children admitted to RCH with high-risk FN, defined as FN occurring in patients with an underlying diagnosis of AML; ALL in dose-intensive treatment phases (induction, consolidation, intensification); lymphoma in induction; ALL or acute lymphoblastic lymphoma (LLy) on Total Therapy Study 17 (TOT17) protocol, or patients with any diagnosis who are within 100 days post allogeneic HSCT.

4.3 Eligibility criteria

Participants will be assigned to a randomised trial intervention only if they meet all of the inclusion criteria and none of the exclusion criteria. Patients can be enrolled repeatedly in the study with multiple, discrete episodes of FN. A new FN episode will be defined as a new fever occurring during a new episode of severe neutropenia (ANC<500 cells/mm^3) and more than 28 days after last randomisation.

4.3.1 Inclusion criteria

Each participant must meet all of the following criteria to be enrolled in this trial:

- Age < 18y at the time of randomisation
- Diagnosis of:
    - AML
    - ALL in dose-intensive phases of induction/re-induction, intensification or consolidation
    - Lymphoma in induction
    - ALL or LLy receiving chemotherapy as per TOT17 protocol
    - Any disease within 100 days of allogeneic or autologous HSCT
- Neutropenia (<500 cells/mm^3)
- Commenced on empiric FN antibiotics (any of piperacillin-tazobactam, cefepime, ceftazidime or ciprofloxacin, +/-vancomycin, +/-amikacin)
- Afebrile (temperature <38.0°C) period for at least 48 hours and no more than 96 hours after at least one temperature measured by axillary or tympanic thermometer (≥38.0°C)
- Clinical stability for at least 48 hours (no conscious state, respiratory rate, respiratory distress, blood pressure, or oxygen saturations in mandatory MET call criteria OR heart rate in the clinical review criteria [> 95th percentile for age] in 48 hours prior to randomisation)

4.3.2 Exclusion criteria

Patients meeting any of the following criteria will be excluded from the trial:

- Prolonged febrile neutropenia (documented daily temperature ≥38.0°C for ≥ 5 days)
- Documented positive blood culture since onset of FN episode and prior to randomisation (microbiologically defined infection)
- Documented other infection requiring antibiotic treatment (clinically or microbiologically defined infection)
- Admitted to the ICU at the time of randomisation
- Within 28 days of last randomisation

4.4 Lifestyle considerations

Not applicable

4.5 Screen failures

Screen failures are defined as participants who consent to participate in the trial but who are found, during the screening procedures, to be ineligible to continue in the trial. As patients may consent before developing FN some consented patients may never develop FN or develop FN and meet an exclusion criteria. They therefore are not randomised and do not receive the intervention. An automated record of all screen failures will be captured electronically by the eMR.

4.6 Recruitment and identification of potential participants

Potential patients will be identified for recruitment in this trial using an alert system within the eMR. The study team will receive an alert notification of a potential participant.
There are three types of patient groups that will be identified for recruitment (i) those with outpatient onset high-risk FN who are admitted to hospital; (ii) inpatients who develop high-risk FN (iii) those high-risk patients (AML, ALL induction/consolidation/DI, lymphoma induction, HSCT d+30, patients receiving chemotherapy on TOT17) admitted to hospital for "neutropenic observation" and placed on the high-risk FN pathway. (Figure 1)

- **Anticipated accrual rate**: Total 111 patients per year (see Table 3) (assuming 20% refusal, 88 per year)
- **Number of sites**: single site.
- **Recruitment setting**: inpatient ward
- **Recruitment strategy**: eMR alert to project team and then verbal consent from study team. A trial information video, accessible by QR code, that summaries the key points from the consent process will be made available to patients and families. Families and patients will be directed to this video if they are (i) admitted for neutropenic observation (ie. admitted with neutropenia and placed on 'high-risk' FN care pathway awaiting fever); (ii) admitted to inpatient ward with FN or (iii) develop FN while an inpatient but not on high-risk FN care pathway. This will mirror the screening process outlined in Section 7.3 'Description of procedures.' The video will also be hosted on the Centre for Health Analytics website that describes the ELSA-FN study (https://www.healthanalytics.org.au/project/elsa-fn/). Posters introducing ELSA-FN and containing a QR code link to the trial information video will be placed in some patient-facing areas (family room and individual patient rooms) within the RCH Children's Cancer Centre at the discretion of the Nurse Unit Manager (see 19.6 Appendix 6).

Participants will not be compensated or provided any incentives for involvement in the trial.

Figure 1. Study overview

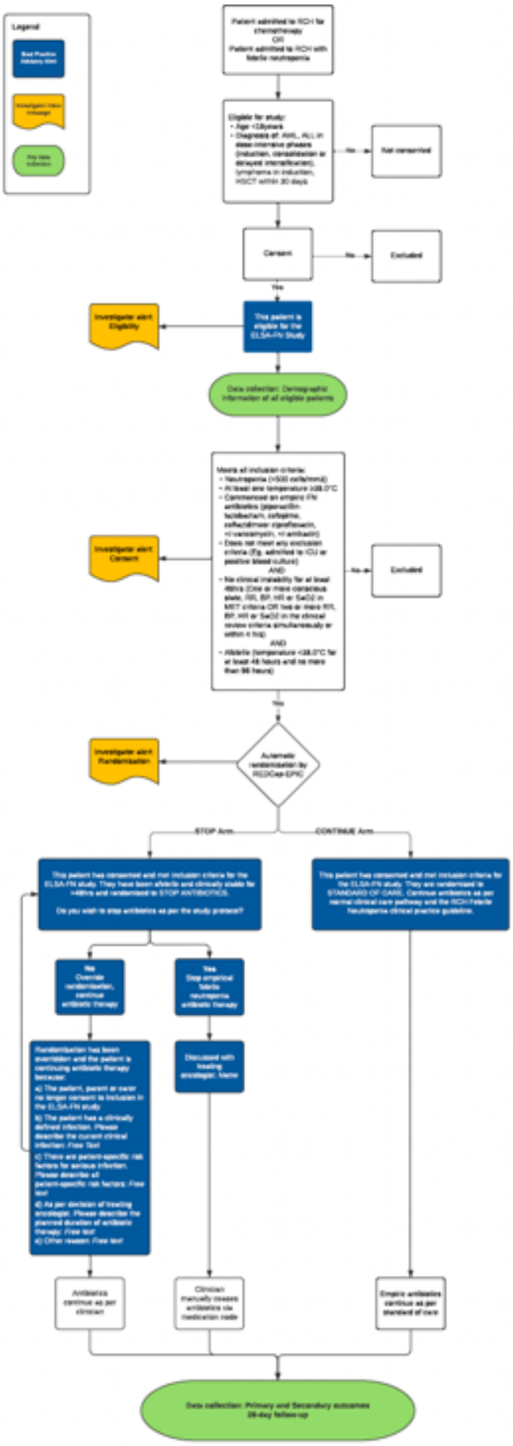

4.7 Consent

Using data that is routinely available in the eMR, potential patients will be identified for study recruitment as outlined above. Patients who have developed FN (but are within 48 hours of initial fever onset), those who are pre-emptively admitted to hospital awaiting fever onset or those planned for HSCT will be consented for the study.

An investigator or delegated member of the trial team will discuss the trial with the parent/legal guardian and, where appropriate, the child/adolescent participant. The consultant/fellow on ward service may perform consent. If the ward service doctor is the patient's primary oncologist every effort will be made for consent to be obtained by another person trained in the study protocol. If the treating consultant is to provide consent they will clarify that the project is voluntary, outside of their routine treatment, and their decision will not influence routine oncology treatment.

To supplement the consent process a trial information video will be made available by QR code for potentially eligible participants. This video will be presented by Dr Gabrielle Haeusler (PI) and Dr Diane Hanna (paediatric oncologist and co-investigator). It will summarise the rationale for the study, describe the randomisation process and provide details about how the trial is embedded.

The investigator/delegated member of the trial team will provide the electronic Participant Information and Consent Form to the parent/legal guardian. This document will describe the purpose of the trial, the procedures to be followed, and the risks and benefits of participation. They will then conduct the informed consent discussion and will check that the parent/guardian comprehends the information provided and answer any questions about the trial.

The parent/legal guardian will be invited to provide written consent using the electronic consent form. Consent will be voluntary and free from coercion. The investigator/delegated member of the trial team who conducted the consent discussion will also document in the eMR that informed consent has been completed. A copy of the consent form will be emailed to the parent/legal guardian.

When all the inclusion/exclusion criteria have been addressed and the eligibility of the participant confirmed, the participant may be randomised to a trial arm. An automated record of all potential participants screened and consented, but not entered into the trial will be captured electronically by the eMR as well as the reasons for not fulfilling all or some of the eligibility criteria. Treating clinicians will be able to over-ride enrolment or randomisation and this data will be collected. All clinicians involved in the study will complete an online learning package to ensure competency in the study protocol.

5 INTERVENTION

5.1 Intervention arms

Short course antibiotics (STOP): Intravenous empiric FN antibiotics (any of: piperacillin-tazobactam, cefepime, ceftazidime, ciprofloxacin, +/-vancomycin, +/-amikacin) will be administered according to current RCH FN clinical practice guideline (CPG) and dosing protocols. Antibiotics will be commenced at onset of FN (as per RCH CPG) and stopped once afebrile and clinically stable for 48 hours.

Standard of care (SOC): Intravenous empiric FN antibiotics (any of: piperacillin-tazobactam, cefepime, ceftazidime, ciprofloxacin, +/-vancomycin, +/-amikacin) will be administered via

current RCH FN CPG and dosing protocols. Antibiotics will be commenced at onset of FN (as per RCH CPG) and continued until resolution of fever, clinical recovery and ANC as determined by the treating clinicians but usually ≥ 500 cells/mm^3

5.2 Intervention(s)

5.2.1 Measurement of participant compliance

Compliance will be measured in the intervention arm as the number of participants ceasing all empiric antibiotics for febrile neutropenia (as per protocol). The duration of antibiotics will also be measured in minutes from the time of eligibility to the final dose administered.

5.2.2 Other

5.2.2.1 Excluded medications and treatments

Not applicable

5.2.2.2 Concomitant therapy

Prescribed antibiotic prophylaxis (ie. ciprofloxacin, TMP-SMX, other), antifungal prophylaxis (ie. fluconazole, voriconazole, other) and granulocyte colony stimulating factor (G-CSF) will be recorded in both arms.

5.2.3 Discontinuation from trial intervention

See Section 7.5.

6 RANDOMISATION AND BLINDING

Study participants will be randomly assigned, in a 1:1 ratio into the 2 study groups. A statistician not directly involved in the analysis of the trial results will prepare the randomisation schedule using block randomisation to maintain balance between treatment arms.
Trial participants and care providers will not be blinded to trial group assignment and beyond. Blinding of trial participants and care providers is not appropriate in this study. To ensure safety of participants on the trial, care providers need to be aware of all antimicrobials that are administered or ceased. This information will inform their treatment decision making if the patient has a new fever, positive blood culture or admission to ICU.

6.1 Concealment mechanism

The randomisation schedule, as detailed above, will be uploaded to the REDCap system at commencement of the study. Randomising investigators will not have access or be able view the randomisation schedule. Project staff with administration access to the REDCap system will not be able to randomise patients.

6.2 Breaking of the trial blind

Not applicable.

7 TRIAL VISITS AND PROCEDURES

7.1 Trial timeline

See Figure 1.

7.2 Schedule of assessments

As an embedded trial, assessment occurs continuously while the patient is admitted to hospital. This includes routine monitoring of vital signs, clinical review as indicated and further blood cultures/intervention as clinically indicated. This information is captured by the eMR and will be used as part of the dataset.

7.3 Description of procedures

Eligible patients will be identified by an eMR alert. Patients will be initially screened for eligibility based on underlying cancer diagnosis. All patients with eligible diagnosis that are (i) admitted for neutropenic observation (ie. admitted with neutropenia and placed on 'high-risk' FN care pathway awaiting fever); (ii) admitted to inpatient ward with FN or (iii) develop FN while an inpatient but not on high-risk FN care pathway will be approached by the study team to discuss participation in the trial. Following documentation of consent, patients will be enrolled and randomised electronically, provided all inclusion and exclusion criteria are fulfilled at the time of randomisation. While every effort will be made to consent patients before or within the first 48 hours of FN onset, patients may be consented, enrolled and randomised up to 96 hours after resolution of fever, provided they continue to meet all inclusion criteria and none of the exclusion criteria. As randomisation occurs after a minimum of 48 hours after FN onset, all patients will initially be managed according to RCH FN and sepsis care pathways.

Eligibility screening, enrolment, randomisation and collection of FN demographic, onset/episode and outcome data will be collected automatically by the Epic eMR system. All clinical definitions and corresponding eMR definitions are available in Appendix 19.4: eMR Definitions.

FN demographic data: (i) patient code; (ii) date of birth; (iii) sex; (iv) tumour type; (v) treatment phase (eg. induction) (vi) date of cancer diagnosis (vii) central venous access (i.e. none, peripherally inserted central catheter, Hickman or portacath); (viii) prescribed antimicrobial prophylaxis (ie. ciprofloxacin, levofloxacin, TMP-SMX, dapsone, pentamidine, amphotericin B, liposomal amphotericin, fluconazole, posaconazole, voriconazole, itraconazole, micafungin, caspofungin) in previous 7 days (ix) prescribed granulocyte colony stimulating factor (G-CSF) in previous 7 days

FN onset/episode data: (i) location at FN onset (outpatient or ward); (ii) date and time of FN onset (inpatient onset) or triage (outpatient onset); (ii) maximum documented temperature (within 4 hours of onset of FN); (iii) ANC at FN onset; (iv) date and time first dose antibiotic (any of piperacillin-tazobactam, cefepime, ceftazidime, ciprofloxacin, vancomycin, amikacin); (v) fluid bolus (≥ 10 ml/kg volume) within 4 hours of FN onset; (vi) blood pressure below and/or heart rate above 95th percentile for age.

Adverse event reporting will be captured automatically in the eMR and monitored by the study team as outlined in section 8 below. Serious Adverse Event (SAE) will include any adverse event/adverse reaction that results in death; is life threatening; requires admission to the ICU,

prolongs hospitalisation, results in re-hospitalisation; or results in persistent or significant disability or incapacity.

7.4 Notes on specific trial visits

7.4.1 Screening

Patients will be screened for inclusion as outlined in Section 7.3 above. Following consent, demographic data will be electronically extracted.

7.4.2 Final trial visit

Final Study Visit (Final Visit, Day 28)

- Patients admitted to hospital at Day 28 assessment: information extracted from eMR
- Patients that are not admitted to hospital at Day 28 assessment: information will be manually extracted from eMR and will include review of all eMR encounters (ie. inpatient and outpatient).

7.5 Treatment discontinuation, participant withdrawals and losses to follow up

7.5.1 Discontinuation of treatment - participant remains in trial for follow up

Participants who discontinue the trial intervention (ie. restart antibiotic during the same neutropenic episode) will remain in the trial, unless consent is withdrawn. The remaining trial procedures should be completed as indicated by the trial protocol.

Participants may discontinue trial intervention for the following reasons:

- Participant / legal guardian request to discontinue trial intervention
- Investigator or the care provider decision to discontinue a participant from the trial intervention if the participant:
    - Experiences a serious or intolerable adverse event such that continued trial intervention would not be in the best interest of the participant
    - Develops, during the course of the trial, symptoms or conditions listed in the exclusion criteria

The investigator may also withdraw all trial participants from the trial intervention if the trial is terminated.

The procedure for transitioning a participant off the trial intervention is as follows: all patients in the STOP antibiotic intervention arm will remain in hospital until ANC count recovery to 200-500 cells/mm^3. They will be placed on the current RCH high-risk FN care pathway. This pathway enables pre-prescribed FN antibiotics to be administered by the nursing team if pre-specific criteria are met (ie. fever ≥38°C or clinical instability) to avoid antibiotic delays. Patients that recommence antibiotics will be defined as discontinuing the trial intervention.

For the safety of all participants ceasing the trial intervention, the protocol-specified safety evaluations will be undertaken to capture new safety events and to assess existing, unresolved safety events. All scheduled follow-ups of trial participants will still occur following intervention discontinuation.

The participant should remain in the trial for scheduled visits for trial assessments (follow-up) per protocol unless consent is withdrawn

7.5.2 Withdrawal of consent - participant withdraws from all trial participation

Participants are free to withdraw from the trial at any time upon their request or the request of their legally acceptable representative. Withdrawing from the trial will not affect their access to standard treatment or their relationship with the hospital and affiliated health care professionals.

For the safety of all participants ceasing trial intervention, reasonable efforts should be made to undertake protocol-specified safety evaluations to capture new safety events and to assess existing, unresolved safety events following withdrawal.

A dedicated Case Report Form (CRF) page will be used to capture the date of participant withdrawal of consent.

7.5.3 Losses to follow-up

A participant will be considered lost to follow-up if discharged from hospital prior to the day 28 assessment and there is no documented encounter in the eMR within 28 days of the final due date assessment.

7.5.4 Replacements

Participants who have been randomised / assigned trial intervention may NOT be replaced.

7.5.5 Trial Closure

A participant is considered to have completed the trial 28 days after randomisation.

The end of the trial is defined as completion of the 28 day follow-up for all patients. At this stage, the Sponsor-Investigator will ensure that all HRECs and RGOs as well as all regulatory and funding bodies have been notified.

This trial may be temporarily suspended or prematurely terminated if there is sufficient reasonable cause. If the trial is prematurely terminated or suspended, the Sponsor-Investigator will promptly inform trial participants, HREC and RGO, the funding (where applicable) and regulatory bodies, providing the reason(s) for the termination or suspension. Circumstances that may warrant termination or suspension include, but are not limited to:

- Determination of an unexpected, significant, or unacceptable risk to participants that meets the definition of a Significant Safety Issue (SSI) (for the definition refer to Section 8.1).
- Insufficient compliance to protocol requirements
- Data that are not sufficiently complete and/or evaluable
- Demonstration of efficacy that would warrant stopping
- Determination that the primary endpoint has been met
- Determination of futility

In the case of concerns about safety, protocol compliance or data quality, the trial may resume once the concerns have been addressed to the satisfaction of the sponsor, HREC, RGO, funding and/or regulatory bodies.

8 SAFETY MONITORING AND REPORTING

8.1 Definitions

Adverse Event (AE): Any untoward medical occurrence in a clinical trial participant in the intervention arm and does not necessarily have a causal relationship with this intervention.

Related Adverse Event (RAE): An adverse event that is judged as having a reasonable causal relationship* with the trial intervention.
Note: *The expression 'reasonable causal relationship' means to convey, in general, that there is evidence or argument to suggest a causal relationship.

Serious Adverse Event (SAE): Any adverse event/adverse reaction that results in:

- death,
- is life threatening*,
- requires hospitalisation, admission to the ICU or prolongation of existing hospitalisation;
- results in persistent or significant disability or incapacity;

Note: *Life-threatening refers to an event in which the participant was at risk of death at the time of the event. It does not refer to an event that hypothetically might have caused death if it were more severe. Medical and scientific judgement will be exercised in deciding whether an adverse event should be classified as serious in other situations. Important medical events that are not immediately life-threatening or do not result in death or hospitalisation but may jeopardise the participant or may require intervention to prevent one of the other outcomes listed in this definition will also be considered serious.

Unexpected and Related Serious Adverse Event (URSAE): An adverse event that is:

- Serious – meets the definition of an SAE (see above)
- Related – resulted from the trial intervention (early stopping of antibiotics)
- Unexpected – the event is not described in the protocol as an expected occurrence

Safety issues (requiring expedited reporting) (see Appendix 2). The following definitions describe additional safety events that require expedited reporting to stakeholders including the Sponsor, Investigators, HREC and local governance office.

Significant Safety Issue (SSI): A safety issue that could adversely affect the safety of participants or materially impact on the continued ethical acceptability or conduct of the trial.
Comment: A SSI is a new safety issue or validated signal considered by the Sponsor in relation to the intervention that requires urgent attention of stakeholders. This may be because of the seriousness and potential impact on the benefit-risk balance of the intervention, which could prompt regulatory action and/or changes to the overall conduct of the clinical trial, including the monitoring of safety and/or the administration of the intervention.

Urgent Safety Measure (USM): A measure required to be taken in order to eliminate an immediate hazard to a participant's health or safety. Note: This is a type of SSI that can be

instigated by either the investigator or sponsor and can be implemented before seeking approval from HRECs or institutions.

8.2 Capturing and eliciting adverse event/reaction information

Adverse events and adverse reactions (non-serious and serious) will be captured from the time of first administration of the intervention (ie. time of randomisation) until 28 days post randomisation.

Whilst in hospital, AE will be captured daily in the participant eMR. If the patient is discharged prior recovery of ANC ≥500 cells/mm^3, the AE will be captured on day 28 review of participant eMR.

8.3 Documentation of AEs

For the purposes of this trial the investigator is responsible for recording any related AEs in the intervention arm, with the following exceptions:

- Conditions that are present at screening and do not deteriorate will not be considered adverse events.
- Abnormalities in blood parameters including renal function (creatinine), liver function tests (LFTs) and Full Blood examination (FBE). These parameters are impacted by underlying chemotherapy and not early stopping of antibiotics

The Related AE will be described in the source documents (Epic eMR) and captured in the electronic Adverse Event Activity within the eMR and will include:

- A description of the AE
- The date of onset, duration, date of resolution
- Severity (mild, moderate or severe – what is the impact on the participant's daily life?)
- Seriousness (i.e. is it a SAE?)
- Any action taken, (e.g. treatment, follow-up tests)
- The outcome (recovery, death, continuing, worsening)
- The likelihood of the relationship of the AE to the trial treatment (Unrelated, Possible, Probable, Definite)

Changes in the severity of an AE will be reported. AEs characterised as intermittent will be documented for each episode. All AEs will be followed to adequate resolution, where possible.

8.4 Assessing the seriousness of a participant's AE

The seriousness of an AE will be assessed by an investigator according to the definition in the preceding section on definitions with the following exception(s):

- Hospitalisation due to progression of disease (ie underlying cancer) will not be considered a SAE for the purposes of this trial.

* The severity and relationship of an AE will be assessed as per the following section.

** The seriousness of an AE will be assessed by an investigator according to the definition in Section 8.1, with the following exceptions:

- Elective surgery planned at the time of enrolment.

8.5 Assessing the relatedness (causality) of a participant's AE

All reportable adverse events will have their relationship to the trial intervention assessed by the investigator who evaluates the adverse event based on temporal relationship and their clinical judgment. The degree of certainty about causality will be graded using the categories below.

- Unrelated: There is no association between the trial intervention and the reported event. AEs in this category do not have a reasonable temporal relationship to exposure to the intervention, or can be explained by a commonly occurring alternative aetiology.
- Possible: The event could have cause or contributed to the AE. AEs in this category follow a reasonable temporal sequence from the time of exposure to the intervention and/or follow a known response pattern to the intervention, but could also have been produced by other factors.
- Probable: The association of the event with the trial intervention seems likely. AEs in this category follow a reasonable temporal sequence from the time of exposure to the intervention and are consistent with the known action of the intervention, known or previously reported adverse events related the intervention, or judgement based on the investigator's clinical experience.
- Definite: The AE is a consequence of administration of the trial intervention. AEs in this category cannot be explained by progression of the condition, concurrent illness or medication, or other factors. Such events may be widely documented as having an association with the intervention or that they occur after rechallenge.

8.6 Assessing the expectedness of a participant's AE

The investigator will be responsible for determining whether an adverse event (AE)in the intervention group is expected or unexpected. An AE will be considered unexpected if the nature, severity, or frequency of the event is not consistent with the risk information previously described for the trial intervention.

The severity of an Adverse Event will be assessed with reference to the most recent version of the Common Terminology Criteria for Adverse Events (CTCAE) produced by the National Cancer Institute (U.S).
https://ctep.cancer.gov/protocoldevelopment/electronic_applications/ctc.htm

8.7 Reporting of safety events

The Principal Investigator/delegate is responsible for recording all safety events in the electronic medical record (Epic) Adverse Event Activity.

The Principal Investigator /delegate is responsible for expedited reporting (within 24 hours of becoming aware of the event) to the Sponsor-Investigator the following safety events:

1. Urgent Safety Measures (USMs)
2. Unexpected and Related Serious Adverse Event (URSAEs)
3. All Serious Adverse Events (SAEs) with the exception of hospitalisation due to progression of disease (ie underlying cancer)

USMs, URSAEs and SAEs should be submitted to the Sponsor-Investigator as soon as possible (but within 24 hours of the first knowledge of the event) using the trial Expedited Safety Report Form.

The Principal Investigator /delegate is also responsible for reporting SSIs, USMs and URSAEs to the research governance office within 72 hours of becoming aware of the event and in accordance with their local governance authorization.

Safety oversight will be under the direction of a Clinical Event Committee (CEC). The CEC will compose an oncologist, infectious diseases physician and statistician, who collectively have experience in the management of paediatrics, biostatistics and the conduct and monitoring of randomised controlled trials. Members of the CEC will be independent of trial conduct. The CEC will convene at the request of the Principal Investigator to review any USMs, URSAEs or SAEs and at least 6-monthly to review all safety events. The CEC will provide its input to the Sponsor-Investigator and where necessary, the HREC.

Sponsor-Investigator Reporting Procedures

The Sponsor-Investigator must assess and categorise the Expedited Safety Reports and report these to the Trial Management Group and the approving HREC in accordance with the NHMRC's 'Safety monitoring and reporting in clinical trials involving therapeutic goods' (November 2016) and any additional requirements of the approving HREC. All safety reports must clarify the impact of the safety event on participant safety, trial conduct and trial documentation.

The Sponsor-Investigator is responsible for the following reporting to the TMG and approving HREC:

1. All SSIs that meet the definition of a USM within 72 hours of becoming aware of the issue.
2. All other SSIs within 15 calendar days of instigating or becoming aware of the issue
3. For SSIs leading to an amendment of trial documentation:
    a. Submit details of the SSI without undue delay and no later than 15 calendar days of becoming aware of the issue.
    b. Submit amendment to the HREC without undue delay.
4. For SSIs leading to temporary halt or early termination of a trial for safety reasons:
    a. Communicate reasons, scope of halt, measures taken, further actions planned without undue delay and no later than 15 calendar days of decision to halt.
    b. For a temporary halt, notify the Site Principal Investigators and approving HRECs when the trial restarts, including evidence that it is safe to do so.

The Sponsor is responsible for providing the additional safety information to the approving HREC:

1. Provide an annual safety report, including a summary of the evolving safety profile of the trial

9 DATA AND INFORMATION MANAGEMENT

9.1 Overview

The Principal Investigator is responsible for storing essential trial documents relevant to data management and maintaining a site-specific record of the location of the site's data management-related Essential Documents.

By using the eMR (Epic), the Source data is attributable, legible (including any changes or corrections), contemporaneous, original, accurate, complete, consistent, enduring and available through secure access. Changes to source data are traceable.

Full details of all processes are provided in a separate trial-level Data Management Plan.

9.2 DATA MANAGEMENT

Data Generation	**9.2.1 Data generation (source data)**<br>In this trial, the following types of data will be collected:<br>• personal identifying information (names, dates of birth, Epic ID)<br>• sensitive information including health data (disease/diagnosis, medical history)<br><br>The source documents for this trial include the RCH eMR, and the signed parent/guardian (and, where applicable participant) information and consent forms.
Data collection – how and by whom will data be generated and collected<br>Use – how and by whom<br>Storage<br>Access – how and by whom, conditions under which access may be granted to others<br>Disclosure – the purpose for which it will be disclosed, to whom?	**9.2.2 Data capture methods and data use, storage, access and disclosure during the trial**<br>Please refer to the ELSA-FN data management plan for full details.<br>**Data collection**<br>Data collected for the study is captured in the eMR. Completion of consent and treatment allocation will be recorded in the eMR by the treating clinician. All clinical outcome and safety measures are collected during standard care within the eMR.<br>**Data storage and access**<br>All data for this trial is stored electronically. The source data is captured within Epic, only personnel with eMR access can view this information. Deidentified datasets created for analysis will contain a unique eMR identifier CSN (contact serial number). Only personnel with Epic access and the ability to search by CSN will be able to identify these patients. Statisticians analysing the data do not have Epic access.<br>**Use of the data**<br>The data will be used as outlined in the data management plan, Statistical Analysis Plan, and as per protocol. Following the completion and analysis of the

	project the data will be retained following the mandatory archive period for long-term use in future research projects.<br><br>**Access to data**<br>The following roles will have access to the data used for this project. The Parkville eMR team will have access to project data in the source eMR system. Table 5 summarises the three types of personal data that will be managed in this project and the roles that will have access to these data.<br><br>**Table 5: Data Access Levels**

Restricted Data Type	Project Roles with Access	Comments
eMR Data in Chronicles	Parkville Clinicians<br><br>RCH Clinician Researchers<br><br>CHA eMR Analysts	All changes and access to clinical data is audited
eMR Reporting Databases	CHA eMR Analysts	
Trial Deidentified Output Data	CHA Embedded Trial Project Officers<br><br>MCTC/CEBU Biostatisticians<br><br>MCRI IT Administrators	

	**Disclosure of data**<br>The study documentation, data and all other information generated will be held in strict confidence. No information concerning the study or the data will be released to any unauthorised third party, without prior written approval of the sponsoring institution. Identifiable information will not be released without written permission of the participant, except as necessary for monitoring by the HREC, Research Governance Office or regulatory agencies
Methods to reduce identification of participants	**9.2.3 Data confidentiality**<br><br><u>Data confidentiality</u>

	Participant confidentiality is strictly held in trust by the Site Principal Investigator, participating investigators, research staff, and the sponsoring institution and their agents.<br>To preserve confidentiality and reduce the risk of identification during collection, analysis and storage of data and information, the following will be undertaken:<br>(1) The number of private/confidential variables collected for each individual has been minimised. The data collected will be limited to that required to address the primary and secondary objectives.<br>(2) The Epic identifier used will be the minimum information required to re-identify that record within the eMR by someone who has access to the eMR. In the ELSA-FN trial, where data is captured at the level of an encounter, the identifier used will be the Epic Contact Serial Number (CSN).<br>(3) Separation of the roles responsible for management of identifiers and those responsible for analysing content. The data will be analysed by the statistician, who will be provided with anonymised data identified only by the unique participant trial ID. *The statistician does not have Epic access.*
Quality assurance	**9.2.4 Quality assurance**<br>Reports will be run on enrolled patients to ensure that the key outcome measures are being captured with the quality required. These reports will be completed weekly for the first 20 patients by a CHA Analyst. At least monthly reviews will then continue while the trial is open to recruitment. The SQL extract (including date, time, timestamp, and interval) will also be run on a monthly basis to ensure that the key outcome measures are being captured as required.<br>Any out-of-range values, invalid dates, or inconsistent data will be raised by the statistician to the TMG who will be able to review this, with assistance from CHA if required.
Storage post-trial<br>ARCHIVE (after trial finished and during archive period)<br>• how will the data be stored post-trial<br>• what is the retention period<br>Disposal –process for safe and secure disposal	**9.2.5 Archiving - Data and document retention**<br>**Archiving**<br>The trial data, information and documents will be securely stored electronically for at least 15 years post-trial completion or until child aged 25 years (whichever is the later). Participant data will only be identified through use of a unique participant trial number/code assigned to the trial participant ("re-identifiable").The sponsor investigator will be the custodian during the archive period. Access to the stored data will be in accordance with 9.2.6 Data Sharing.

	**Destruction**<br>The source data for patients is collected routinely in the eMR. As this is routinely collected in clinical care, this won't be destroyed after the minimum retention periods. Data files created for the study may be destroyed after a minimum of 25 years post trial completion.
Data sharing – plans for permitting re-use of data both internal and external?	**9.2.6 Data sharing**<br>Beginning 12 months following analysis and article publication, the following will be made available long-term for use by future researchers from a recognised research institution whose proposed use of the data has been ethically reviewed and approved by an independent committee and who accept MCRI's conditions for access:<br>• Individual participant data that underlie the results reported in this article after de-identification (text, tables, figures and appendices)<br>• Trial protocol, Statistical Analysis Plan, PICF
Long-term custodianship (after archive period finished)	**Long-term custodianship (after archive period finished)**<br>After the archive period, the data will be anonymised for preservation to reduce the risk of re-identification. The principal investigator will be the long-term data custodian following the archive period.
Sample management	Not applicable. A biobank will not be generated.

10 TRIAL OVERSIGHT

10.1 Governance structure

10.1.1 Trial Management Group (TMG)

The Site Principal Investigator is responsible for supervising any individual or party to whom they have delegated tasks at the trial site. They will provide continuous supervision and documentation of their oversight. To meet this GCP requirement, a small group will be responsible for the day-to-day management of the trial and will include at a minimum the Site PI, project manager/research nurse/trial coordinator, and PhD student. The group will closely review all aspects of the conduct and progress of the trial, ensuring that there is a forum for identifying and addressing issues. Meetings must be minuted with attendees listed, pertinent emails retained and phone calls documented.

10.1.2 Trial Steering Committee (TSC)

A TSC has been established to provide expert advice and overall supervision, and ensure that the trial is conducted to the required standards. The TSC will meet at least annually, with more frequent meetings as needed, and will work to a Terms of Reference.

10.1.3 Safety Monitoring

Safety oversight will be under the direction of a Clinical Event Committee (CEC). It will compose an oncologist, infectious diseases physician and statistician, who, collectively, have experience in the management of paediatrics, biostatistics and the conduct and monitoring of randomised controlled trials. Members of the CEC will be independent of trial conduct. The CEC will meet as required, but at least 6-monthly, to review the SAEs. The CEC will provide its input to the Sponsor-Investigator and where necessary, the HREC.

10.2 Quality Control and Quality Assurance

Both the Sponsor-Investigator and Site Investigator have responsibilities in relation to quality management.

The Sponsor-Investigator will develop SOPs that identify, evaluate and control risk for all aspects of the trial, e.g. trial design, source data management, training, eligibility, informed consent and adverse event reporting. The Sponsor-Investigator will also implement quality control (QC) procedures, which will include the data entry system and data QC checks. Any missing data or data anomalies will be communicated to the site(s) for clarification/resolution.

In the event of non-compliance that significantly affects human participant protection or reliability of results, the Sponsor-Investigator will perform a root cause analysis and corrective and preventative action plan (CAPA).

11 STATISTICAL METHODS

11.1 Sample Size Estimation

When the sample size in each group is 147, a two-group large-sample normal approximation test of proportions with a one-sided 2.5% significance level will have 80% power to reject the null hypothesis that the test and the standard are not equivalent (the difference in proportions, $\pi_1 - \pi_0$, is 0.15 or farther from zero in the same direction) in favour of the alternative hypothesis that the proportions in the two groups are equivalent, assuming that the expected difference in proportions is 0 and the proportion in the standard group is 0.3. This sample size assumes that all participants are independent, however, some participants can be enrolled in the trial more than once. This can be accommodated in the sample size calculation by a design effect. We calculated the design effect to be 1.06. This is calculated using the formula in Yelland *et al*[27] assuming that 50% of enrolments will be enrolled only once, and the rest would be enrolled more than once, that the proportion in the intervention group was 0.3 and 0.3 to 0.33 in the control group. ρ is assumed to be 0.3. When we apply the design effect to the sample size calculated the final sample size is 156 per arm or 312 in total.

11.2 Population to be analysed

The following analysis populations are defined.

- Intention to treat population (ITT): Includes any participant randomised into the trial, regardless of whether they received the intervention.
- Per-protocol population (PP): Includes any participant who was randomised and received the intervention as per the protocol and fulfilled all protocol required assessments

11.2.1 Handling of missing data

If a large amount of study data is missing, multiple imputation methods will be used to handle the missing data appropriately. These methods will be described in detail in the statistical analysis plan.

11.3 Methods of analysis

The incidence of the primary outcome, unfavourable clinical course, will be calculated in each treatment arm with a 95% confidence interval. The outcome will be modelled using a generalised linear mixed model (GLMM) with a logit link function (for the binary outcome) and a Bernoulli distribution. This model will be adjusted for baseline covariates. Patients will be included as a random effect, to consider the fact that the same child can be enrolled more than once. This model will be used to calculate the difference in proportions between the two randomised treatment arms, with a 95% confidence interval.

The non-inferiority margin was set at 15%. If the upper limit of the 2-sided 95% confidence interval is below 15%, we will declare the STOP arm as non-inferior to the standard of care arm. The non-inferiority margin of 15% is higher than that used in standard drug-drug trials but appropriate in this study. This margin was chosen as it was shown to be clinically acceptable to a representative sample of RCH oncology clinicians. We also discussed validity of this non-inferiority margin with an international consortium of paediatric febrile neutropenia researchers. While a 15% increase in the least common complications of febrile neutropenia (death, ICU admission, physiological instability) would not be acceptable, this is acceptable for the most common complication (recurrence of fever). According to national PICNICC data 30% of patients are projected to have recurrence of fever in the SOC arm and it was deemed clinically acceptable for up to 45% of patients in the STOP arm (margin +15%) to have recurrence of fever.

All binary secondary outcomes will be analysed in the same way as described for the primary outcome (recurrence of fever, clinical instability, admission to ICU, mortality, new positive blood culture, readmission to hospital, development of C. difficile infection, development of an antibiotic resistant infection or colonisation).

The continuous secondary outcomes (duration of neutropenia, total antibiotic duration and total hospital length of stay) will be analysed using a generalised linear mixed model (GLMM) with an identity link function and a normal distribution. This model will be adjusted for baseline covariates and stratification variables. Patient will be included as a random effect, to consider the fact that the same child can be enrolled more than once. This model will be used to calculate the difference in means between the two randomised treatment arms, with a

95% confidence interval. Variables that are skewed, will be analysed using non-parametric methods (Mann Whitney test) or zero-inflated models, as appropriate.

The study is powered for the Intention-to-treat analysis, with the per protocol analysis as a supporting sensitivity analysis. The trial is not powered for the per protocol analysis. The amount of crossover between treatment arms will be assessed and reported. If the amount of crossover is large, additional statistical methods that can deal with non-randomised comparisons, such as g-methods or inverse probability weighting will be considered. A statistical analysis plan will be written prior to database lock where the details of statistical analysis will be provided.

11.4 Interim Analyses

Not applicable

12 ETHICS AND DISSEMINATION

12.1 Research Ethics Approval & Local Governance Authorisation

This protocol and the informed consent document and any subsequent amendments will be reviewed and approved by the human research ethics committee (HREC) prior to commencing the research. A letter of protocol approval by HREC will be obtained prior to the commencement of the trial, as well as approval for other trial documents requiring HREC review.

12.2 Amendments to the protocol

This trial will be conducted in compliance with the current version of the protocol. Any change to the protocol document or Informed Consent Form that affects the scientific intent, trial design, participant safety, or may affect a participant's willingness to continue participation in the trial is considered an amendment, and therefore will be written and filed as an amendment to this protocol and/or informed consent form. All such amendments will be submitted to the HREC, for approval prior to being implemented.

12.3 Protocol Deviations and Serious Breaches

All protocol deviations will be recorded in the participant record (source document) must be reported to the Principal Investigator, who will assess for seriousness.

Those deviations deemed to affect to a significant degree rights of a trial participant or the reliability and robustness of the data generated in the clinical trial will be reported as serious breaches. Reporting will be done in a timely manner. Sponsor-Investigator to review and submit to the approving HREC within 7 days.

Where non-compliance significantly affects human participant protection or reliability of results, a root cause analysis will be undertaken and a corrective and preventative action plan prepared.

Where protocol deviations or serious breaches identify protocol-related issues, the protocol will be reviewed and, where indicated, amended.

13 CONFIDENTIALITY

Participant confidentiality is strictly held in trust by the participating investigators, research staff, and the sponsoring institution and their agents. This confidentiality is extended to cover testing of biological samples in addition to the clinical information relating to participating participants.

The trial protocol, documentation, data and all other information generated will be held in strict confidence. No information concerning the trial or the data will be released to any unauthorised third party, without prior written approval of the sponsoring institution. Authorised representatives of the sponsoring institution may inspect all documents and records required to be maintained by the Investigator, including but not limited to, medical records (office, clinic or hospital) for the participants in this trial. The clinical trial site will permit access to such records.

All laboratory specimens, evaluation forms, reports and other records that leave the site will be identified only by the Participant Identification Number (SID) to maintain participant confidentiality.

Clinical information will not be released without written permission of the participant, except as necessary for monitoring by HREC or regulatory agencies.

14 PARTICIPANT REIMBURSEMENT

No participant reimbursements will be offered

15 FINANCIAL DISCLOSURE AND CONFLICTS OF INTEREST

No conflicts of interest or financial disclosures

16 DISSEMINATION AND TRANSLATION PLAN

The sponsor-investigator holds primary responsibility for publication of the results in a peer-review journal. Trial results will also be disseminated through presentation at national and international meetings and to the Royal Children's Hospital Oncology Group. The results will also inform guidelines for the management of febrile neutropenia.

17 ADDITIONAL CONSIDERATIONS

Nil

18 REFERENCES

1. 1. Haeusler GM, Sung L, Ammann RA, et al: Management of fever and neutropenia in paediatric cancer patients: room for improvement? Curr Opin Infect Dis 28:532-8, 2015

2. Levene I, Castagnola E, Haeusler GM: Antibiotic-resistant Gram-negative Blood Stream Infections in Children With Cancer: A Review of Epidemiology, Risk Factors, and Outcome. Pediatr Infect Dis J 37:495-498, 2018

3. Haeusler GM, Mechinaud F, Daley AJ, et al: Antibiotic-resistant Gram-negative bacteremia in pediatric oncology patients--risk factors and outcomes. Pediatr Infect Dis J 32:723-6, 2013

4. Haeusler GM, Thursky KA, Mechinaud F, et al: Predicting Infectious ComplicatioNs in Children with Cancer: an external validation study. Br J Cancer 117:171-178, 2017

5. Lehrnbecher T, Robinson P, Fisher B, et al: Guideline for the Management of Fever and Neutropenia in Children With Cancer and Hematopoietic Stem-Cell Transplantation Recipients: 2017 Update
. J Clin Oncol 35:2082-2094, 2017

6. Haeusler GM, Thursky KA, Slavin MA, et al: Risk stratification in children with cancer and febrile neutropenia: a national, prospective, multicentre validation of nine clinical decision rules. EClinicalMedicine 18:100220, 2020

7. Freifeld AG, Bow EJ, Sepkowitz KA, et al: Clinical practice guideline for the use of antimicrobial agents in neutropenic patients with cancer: 2010 update by the infectious diseases society of america. Clin Infect Dis 52:e56-93, 2011

8. Klastersky J, de Naurois K, Rolston KV, et al: MANAGEMENT OF FEBRILE NEUTROPAENIA: ESMO CLINICAL PRACTICE GUIDELINES. Ann Oncol 27:111-118, 2016

9. National Institute for Health and Care Excellence (NICE) guideline: Neutropenic sepsis: prevention and management in people with cancer (updated Jan 2020). www.nice.org.uk/guidance/cg151, 2012

10. Averbuch D, Orasch C, Cordonnier C, et al: European guidelines for empirical antibacterial therapy for febrile neutropenic patients in the era of growing resistance: summary of the 2011 4th European Conference on Infections in Leukemia. Haematologica 98:1826-35, 2013

11. Stern A, Carrara E, Bitterman R, et al: Early discontinuation of antibiotics for febrile neutropenia versus continuation until neutropenia resolution in people with cancer. Cochrane Database Syst Rev 1:CD012184, 2019

12. Aguilar-Guisado M, Espigado I, Martin-Pena A, et al: Optimisation of empirical antimicrobial therapy in patients with haematological malignancies and febrile neutropenia (How Long study): an open-label, randomised, controlled phase 4 trial. Lancet Haematol 4:e573-e583, 2017

13. Santolaya ME, Villarroel M, Avendaño LF, et al: Discontinuation of antimicrobial therapy for febrile, neutropenic children with cancer: a prospective study. Clin Infect Dis 25:92-7, 1997

14. Santolaya ME, Alvarez AM, Acuña M, et al: Efficacy and safety of withholding antimicrobial treatment in children with cancer, fever and neutropenia, with a demonstrated viral respiratory infection: a randomized clinical trial. Clin Microbiol Infect 23:173-178, 2017

15. Koenig C, Schneider C, Morgan JE, et al: Association of time to antibiotics and clinical outcomes in patients with fever and neutropenia during chemotherapy for cancer: a systematic review. Support Care Cancer 28:1369-1383, 2020

16. Burns C, Borello E, Mechinaud F, et al: Time to first dose antibiotic in high-risk paediatric inpatients with cancer and febrile neutropenia: A quality improvement project. ANZCHOG ASM, 2016 (poster), 2016

17. Haeusler GM, De Abreu Lourenco R, Clark H, et al: Diagnostic Yield of Initial and Consecutive Blood Cultures in Children With Cancer and Febrile Neutropenia. J Pediatric Infect Dis Soc, 2020

18. Orme LM, Babl FE, Barnes C, et al: Outpatient versus inpatient IV antibiotic management for pediatric oncology patients with low risk febrile neutropenia: a randomised trial. Pediatr Blood Cancer 61:1427-33, 2014

19. Cheng S, Teuffel O, Ethier MC, et al: Health-related quality of life anticipated with different management strategies for paediatric febrile neutropaenia. Br J Cancer 105:606-11, 2011

20. McMullan BJ, Haeusler GM, Hall L, et al: Aminoglycoside use in paediatric febrile neutropenia - Outcomes from a nationwide prospective cohort study. PLoS One 15:e0238787, 2020

21. Haeusler GM, Levene I: Question 2: what are the risk factors for antibiotic resistant Gram-negative bacteraemia in children with cancer? Arch Dis Child 100:895-8, 2015

22. Wolf J, Tang L, Flynn PM, et al: Levofloxacin Prophylaxis During Induction Therapy for Pediatric Acute Lymphoblastic Leukemia. Clin Infect Dis 65:1790-1798, 2017

23. Haeusler GM, Phillips RS, Lehrnbecher T, et al: Core outcomes and definitions for pediatric fever and neutropenia research: a consensus statement from an international panel. Pediatr Blood Cancer 62:483-9, 2015

24. Haeusler GM, Phillips RS, Lehrnbecher T, et al: Core outcomes and definitions for pediatric fever and neutropenia research: a consensus statement from an international panel. Pediatric Blood & Cancer 62:483-9, 2015

25. Curran GM, Bauer M, Mittman B, et al: Effectiveness-implementation hybrid designs: combining elements of clinical effectiveness and implementation research to enhance public health impact. Med Care 50:217-26, 2012

26. Sekhon M, Cartwright M, Francis JJ: Development of a theory-informed questionnaire to assess the acceptability of healthcare interventions. BMC Health Serv Res 22:279, 2022

27. Hennink M, Kaiser BN: Sample sizes for saturation in qualitative research: A systematic review of empirical tests. Soc Sci Med 292:114523, 2022

19 APPENDICES

19.1 Appendix 1: Division of sponsor responsibilities between sponsor and sponsor-investigator

**Sponsor-Investigator:**	A/Prof Gabrielle Haeusler	
**Responsibility**	**Sponsor**	**Sponsor-Investigator**
Ensure a peer review/independent expert review has demonstrated that the trial proposal is worthwhile and is of high scientific quality.	X	
Ensure the Sponsor-Investigator has adequate procedures in place for all key trial management activities	X	
Assign an overall risk category based on type of intervention	X	
Ensure that the Sponsor-Investigator has the necessary expertise and experience to conduct the trial	X	
Ensure that the Sponsor-Investigator has the resources needed to complete the trial successfully or that plans are in place to raise additional funds.	X	
Confirm provision of insurance and indemnity for the trial and trial related staff as well as measures for participant compensation for trial related injury	X	
Ensure all the roles and responsibilities for the clinical trial are delegated, agreed and documented appropriately	X	
Oversee/sign-off all contract negotiations with external providers (e.g. external lab facilities; pharmaceutical companies for supply of investigational product,)	X	
Ensure the protocol (or other document) details appropriate monitoring and management plans commensurate to the risk and complexity of the trial	X	
Maintain oversight to include audit, where applicable	X	
Ensure that the trial is based on a thorough review of scientific literature including whether any relevant systematic review exists.		X
Secure funding and/or confirm sufficient resources are available to conduct the trial (e.g. trial subjects, time, staff, facilities, finances) or put in place plans to raise additional funds.		X
Ensure that trials are registered on clinical.trials.gov, ANZCTR or other appropriate registry before first patient is enrolled and that appropriate plans for the dissemination of trial findings are in place		X
Unless delegated to a third party, undertake/oversee the design, conduct and reporting of the trial with support from all relevant specialist staff (e.g. statistician, research methodologist) including the development of a protocol that is compliant with international standards including the SPIRIT Statement.		X
Ensure a trial risk assessment has been carried out and proportionate trial management and monitoring plans are in place		X
Develop/endorse an appropriate strategy for independent trial oversight (e.g. Trial Management Group, Trial Steering Committee, Data Safety Monitoring Board)<br>If a Data Safety Monitoring Board is not warranted, ensure alternative mechanisms for ongoing safety monitoring are in place		X
Document trial specific delegation of duty on a Staff Signature and Delegation Log		X
Confirm each member of the trial team are aware of their trial-related duties		X
Ensure the development of all relevant trial documentation (e.g. protocol, Participant Information and Consent Form and Case Report Form)		X
Clearly identify the reference safety information to allow identification of expectedness of adverse events		X
Oversee the set-up of a clinical trial database		X

Ensure all trial approvals and notification are in place before the trial commences (e.g. HREC, SSA, TGA)		X
Ensure relevant agreements/signatories from service departments supporting the trial (e.g. laboratories, radiology) are obtained		X
Ensure arrangements are in place for the effective financial management of the trial		x
Prepare and submit amendments to the trial		X
Implement procedures to ensure the collection of high quality and accurate data		X
Oversee the set-up and maintenance of a Trial Master File		X
Ensure safety reporting and monitoring for the trial complies with the requirements of the NHMRC Guidance for Safety Monitoring and confirm and execute any sponsor reporting responsibilities that are delegated		X
Submit annual report(s) to the HREC and Research Office in accordance with Australian Guidance and local requirements		X
Report suspected serious breaches of GCP/protocol to the HREC and Research Office in accordance with the NHMRC Guidance		X
Notify HREC, Research Office and other relevant bodies of the completion of the trial		X
Produce all necessary reports to funders and others		X
Disseminate trial findings through publication/dissemination of trial results where applicable, following the CONSORT Statement		X
Fulfil commitments to trial participants, such as providing information about the outcome(s) of the trial, re-obtaining consent if required due to change in risk-benefit ratio (of investigational medicinal product) or change in protocol procedures		X
Ensure all trial data (including the Trial Master File) and materials, are archived appropriately and retrievable for audit purposes		X
Maintain trial registration record in accordance with the registry's requirements		X

19.2 APPENDIX 2: Significant Safety Issues (SSI) - some examples

Examples below have been extracted from the NHMRC's "Safety monitoring and reporting in clinical trials involving therapeutic goods" (November 2016)

- a serious adverse event that could be associated with the trial procedures and that requires modification of the conduct of the trial
- a hazard to the patient population, such as lack of efficacy of an intervention used for the treatment of a life-threatening disease
- a temporary halt/termination of a trial for safety reasons
- a recommendation from the Data Safety Monitoring Board relevant to the safety of participants

To these could be added:

- an unexpected and related serious adverse event that requires modification of the conduct of the trial

The examples below have been extracted from the TGA's "Pharmacovigilance responsibilities of medicine sponsors: Australian recommendations and requirements" Version 2.0, September 2017 and some may be applicable to non-drug, biologic or device interventions:

- changes in the nature, severity or frequency of known related adverse events which are medically significant
- safety issues due to misinformation in the protocol that may impact the safety of the intervention
- an unusual and significant lack of efficacy occurring in or outside Australia that may have implications for public health
- a signal of significant hazard to public health

19.3 APPENDIX 3: Expedited Safety Report Form

EXPEDITED SAFETY REPORT FORM

Reporting requirement: All sites to report to Sponsor-Investigator all SAEs*, URSAEs and USMs within 24 hours of **trial** staff becoming aware of the event.
**Except those identified in the protocol as not needing immediate reporting*

**HREC Reference #**	74690
**Project title**	Early Versus Late Stopping of Antibiotics in Febrile Neutropenia

**Section A: To be completed by the Local Site**		
**Site:**	Royal Children's Hospital	
**Local Site Principal Investigator:**	A/prof Gabrielle Haeusler	
**Participant Enrolment OR Randomisation No.:**		
**Date the safety event occurred:**		
**Date Local Site Principal Investigator became aware of the safety event:**		
**Participant's date of birth, age and weight:**		
**Event description and management:**		
**Event outcome (synopsis):**		
**Trial phase** *(amend to reflect protocol)*	☐ Screening<br>☐ Intervention phase<br>☐ Follow Up phase	
**Relationship to the trial intervention**	☐ Unrelated<br>☐ Unlikely to be related<br>☐ Possibly related<br>☐ Probably related	
**Expectedness (for SAEs only complete for those that are probably/possibly related):**	☐ Not applicable<br>☐ Expected<br>☐ *Unexpected<br>*Report URSAE to local RGO within 72 hours of becoming aware of event	
**Was an Urgent Safety Measure (USM) instigated?**<br>*A measure required to be taken in order to eliminate an immediate hazard to a participant's health or safety.*	☐ * Yes ☐ No<br>*Report USM to local RGO within 72 hours of becoming aware of event	
Name and Signature (of local PI or delegate)		Date

Section B: To be completed by the Sponsor-Investigator only	
**Is this event a Significant Safety Issue (SSI)?**<br>*A safety issue that could adversely affect the safety of participants or materially impact on the continued ethical acceptability of the trial and may require action such as the reporting of an urgent safety measure (USM), submission of a protocol amendment, a temporary halt or early termination of a trial.*	*☐ Yes ☐ No<br><br>* Report to HREC and all site PIs within 15 days of becoming aware of event
**Is this event an Urgent Safety Measure (USM)?**<br>*A measure required to be taken in order to eliminate an immediate hazard to a participant's health or safety.*	* ☐ Yes ☐ No<br><br>*Report to HREC and all site PIs within 72 hours of becoming aware of event
**Does the <u>protocol</u> require amending as a result of this safety event?**<br>(If Yes, submit an amended protocol to approving HREC)	☐ Yes ☐ No
**Do the <u>participant information statements</u> require amending as a result of this safety event?**<br>(If Yes, submit a**n amendment** request to approving HREC and RGOs with the amended forms)	☐ Yes ☐ No
**Is a temporary halt or early termination of the trial required as a result of this safety event?**<br>(If Yes, ensure actions are taken within 15 days of decision to halt)	☐ Yes ☐ No
Name and Signature (of Sponsor-Investigator)	Date

Please email one signed copy to the Sponsor–Investigator (Gabrielle.haeusler@rch.org.au) and retain the signed original in the Site Investigator File

19.4 APPENDIX 4: eMR Definitions

Clinical and electronic medical record study definitions		
	**Clinical definition**	**eMR definition**
**Fever**	Single temperature ≥ 38.0 °C[28]	Single temperature ≥ 38.0 °C
**Neutropenia**	Absolute neutrophil count (ANC) < 0.5 x 10^9/L [23]	Absolute neutrophil count (ANC) < 0.5 x 10^9/L or total white cell count (WCC) <1 x 10^9/L
**Febrile neutropenia onset**	First onset of fever in the setting of neutropenia	Fever within 24h of recorded neutropenia (pre- or post-fever) or recorded triage time if presenting to the emergency department with FN
**High risk FN**	FN occurring in patients at high risk of infection or adverse outcome including patients with diagnosis of AML; ALL induction, consolidation of delayed intensification phases; lymphoma in indication; on TOT17 protocol; or in first 100 days of HSCT	FN occurring in patients with any of:<br>AML (any phase)<br>ALL (induction/re-induction, intensification or consolidation treatment phase)<br>Lymphoma (induction)<br>ALL or LLy receiving TOT17 (induction, consolidation or continuation)<br>HSCT day +30
**End of FN episode**	Afebrile for > 48 hours, no clinical instability for >48 hours and recovery of ANC to ≥0.5 x 10^9/L[6]	No fever recorded for > 48 hours and ANC documented ≥0.5 x 10^9/L
**Positive blood culture prior to randomisation**	A blood culture that is taken after FN onset that returns a positive result prior to randomisation	A blood culture that is taken after FN onset that becomes culture-positive with any organism prior to randomisation
**Admission to ICU prior to randomisation**	Admission to ICU for any duration after FN onset and prior to randomisation	Not admitted to ICU at the time of randomisation
**Admission to ICU for organ support**	Admission to ICU in which management includes any of the following:<br>• Inotropes/vasopressors<br>• Renal replacement therapy<br>• Invasive or non-invasive ventilation	Admission to ICU in which management includes any of the following:<br>• Inotropes/vasopressors: adrenaline, noradrenaline, dopamine, dobutamine, vasopressin, milrinone<br>• Renal replacement therapy: continuous renal replacement therapy, intermittent haemodialysis, peritoneal dialysis<br>• Invasive ventilation: mechanical ventilation<br>• Non-invasive ventilation: bi-level positive airway pressure (BiPAP), conitinuous positive airway pressure

**Clinical Stability**	Physiologically stable at the time of randomisation.	-No MET call note within previous 48hrs (conscious state, blood pressure, heart rate, respiratory rate, respiratory effort or oxygen saturation have not breeched age-based MET criteria) AND<br>-Not meeting clinical review criteria for heart rate within previous 48hrs (HR >95th percentile for age) AND<br>-Not in clinical review criteria (conscious state, blood pressure, heart rate, respiratory rate or oxygen saturation) at the time of randomisation
**New fever episode**	A new fever after an afebrile period of ≥48h	A new fever after an afebrile (temperature <38°C) period of ≥48h
**New FN episode**	A new fever occurring during a new episode of neutropenia (ANC<500 cells/mm^3) and more than 28 days after last randomisation.[6]	Neutropenia and a new fever occurring after the 'end of FN episode' and more than 28 days after last randomisation
**Prolonged FN**	Neutropenia and documented daily temperature ≥38.0°C for ≥ 5 days	Neutropenia and documented temperature ≥38.0°C every day for ≥5 days
**Unfavourable clinical course**	Any of the following occurring after randomisation and during the same period of neutropenia (ANC <500 cells/mm^3):	Any of the following occurring after randomisation and during the same period of neutropenia (ANC <500 cells/mm^3):
	Recurrence of fever ≥38.0 °C after afebrile period of 48h	Documented temperature ≥38.0 °C after ≥48h period of temperature <38.0°C
	Clinical instability	-One or more vital signs (conscious state, blood pressure, heart rate, respiratory rate or oxygen saturation) in age-based MET criteria, OR<br>-Two or more vital signs (blood pressure, heart rate, respiratory rate or oxygen saturation) simultaneously (+/- 4 hours) in age-based clinical review criteria
	Admission to intensive care unit (ICU)	Admission to ICU (Rosella Ward) for any reason
	New positive blood culture	Blood culture collected after randomisation and during same period of neutropenia that becomes culture-positive (with any organism)

	Death	Death (from any cause)
**Antibiotic duration**	Duration of all antibiotics (excluding PJP and febrile neutropenia prophylaxis) and calculated as days of therapy	Date time of first dose antibiotic to date and time of last antibiotic dose (any antibiotic with the exception of prophylaxis using TMP-SMX, ciprofloxacin or levofloxacin)
**Total in-hospital LOS**	Duration of in-hospital length of stay calculated from in-hospital admission date and time to discharge or transfer from in-hospital ward	Date and time of in-hospital admission to date and time of discharge or transfer from in-hospital ward
**Total hospital LOS**	Duration of in-hospital length of stay calculated from randomisation date and time to discharge from hospital ward or HITH	Date and time of randomisation to date and time of hospital discharge (including from HITH)
**Unplanned readmission**	Readmission to hospital within 28 days of randomisation	Unplanned readmission to hospital (inpatient ward) within 28 days of randomisation
**Duration of neutropenia**	Days of neutropenia from FN onset to recovery of ANC >/= 0.5	Date and time of fever onset to date and time of first ANC >/=0.5

**Cause of fever**	Microbiologically defined infection (MDI) – an infection that is clinically detectable and microbiologically proven.<br><br>Bacteraemia - a recognized pathogen cultured from one or more blood cultures (includes viridans group streptococci in the setting of concomitant mucosal barrier injury). Common commensals should be cultured from two or more blood cultures drawn on separate occasions. Where only a single blood culture is taken, and in the presence of a long term vascular catheter, common commensals cultured once may be included if an alternative source of infection is not identified. (Blood cultures drawn from different sites including different venepunctures or different lumens of the same central line, should undergo separate decontamination and are therefore considered drawn on “separate occasions”.)[24]<br>-Clinically defined infection (CDI) – an infection that is clinically detectable but no pathogen is identified<br>-Fever without a focus – fever within a documented MDI or CDI	Will require manual allocation following chart review.
**Time to first dose antibiotic**	Time to first dose antibiotic calculated for initial FN episode and clinical failure (starting new antibiotic)	Date and time from first fever and/or clinical instability to first dose antibiotic.
**28 days mortality**	Death within 28 days of randomisation	Died within 28 days of randomisation
**Prescribed antimicrobial prophylaxis**	Prescribed antimicrobial prophylaxis in 7 days prior to randomisation and/or within 28 days of randomisation.	Any of the following prescribed in 7 days prior to randomisation and/or within 28 days of randomisation:<br>ciprofloxacin, levofloxacin, TMP-SMX, dapsone, pentamidine, amphotericin B, liposomal amphotericin, fluconazole, posaconazole, voriconazole, itraconazole, micafungin, caspofungin

**Antibiotic resistant infection or colonisation**	Antibiotic resistant (MRSA, ESBL-producing enterobacterales, CRE, VRE) infection or colonisation within 28 days of randomisation	Any of the following identified from sterile site culture (blood, urine, cerebrospinal fluid, peritoneal fluid, synovial fluid) or screening swab/stool:<br>• Methicillin-Resistant *Staphylococcus aureus* (MRSA): Staphylococcus aureus reported resistant to oxacillin<br>• Extended Spectrum Beta Lactamase-producing enterobacterales (ESBL): *Escherichia coli, Klebsiella species., Enterobacter spp., Morganella spp., Providencia spp*. or *Proteus spp.* in which a transmissible ESBL enzyme or plasmid-mediated AmpC has been reported.<br>• Carbapenemase-Producing Enterobacterales (CPE): *Escherichia coli, Klebsiella species., Enterobacter spp., Morganella spp., Providencia spp*. or *Proteus spp.* reported as resistant to meropenem<br>• Vancomycin Resistant Enterococci (VRE): *Enterococcus faecalis* or *Enterococcus faecium* reported resistant to vancomycin.<br>• Multidrug-resistant Pseudomonas aeruginosa (MRPAER): P. aeruginosa resistant to at least 2 or more of gentamicin, ciprofloxacin or a beta-lactams (e.g. piperacillin, ceftriaxone, ceftazidime, meropenem)
**Clostridium difficile infection**	Diarrhoea (usually defined as 3 or more loose stools in a 24 hour period), ileus, toxic megacolon or pseudomembranous colitis (identified by colonoscopy) with positive stool test for toxin-producing *C. difficile*.	Positive laboratory test result for *C. difficile* toxin A and /or B tested on an unformed (diarrhoea) stool specimen.
**Clinically documented infection (CDI)**	An infection that is clinically diagnosed but no pathogen cultured	Will require manual allocation following chart review.

19.5 APPENDIX 5: *ELSA-Impact* – Research methodology for antibiotic prescribing focus groups and cost-effectiveness

This exploratory qualitative component will examine the complex factors influencing antibiotic decision-making and develop a model of factors influencing prescribing by clinicians caring for children with cancer. It will also look at factors among patients and parents or carers

Methodology. An effectiveness-implementation hybrid design (Type 1)[25] will be used to primarily test the clinical efficacy and safety of the intervention (ie the ***ELSA-FN*** trial) whilst simultaneously gathering information on the implementation of the intervention in a real-world clinical setting. In ***ELSA-Impact*** this will include determining the acceptability and the barriers and enablers to the intervention, which will generate strategies to inform future implementation and scaling. Quantitative and qualitative data will be collected according to the 'Theoretical Framework of Acceptability (TFA) that encompass 7 domains (affective attitude, burden, ethicality, coherence, opportunity costs, perceived effectiveness, self-efficacy).'[26]

Exploratory semi-structured focus groups will be conducted with prescribing clinicians (registrar, fellow, junior consultant and senior consultant level) as well as nursing and pharmacy staff caring for children with FN at RCH. These meetings will focus on attitudes towards short-course antibiotics and perceived barriers and enablers to adoption of this model established. Focus group meetings with patients, parents and carers who have been included in the primary study (ELSA-FN) will explore attitudes to antibiotic use during FN including factors influencing preferences for early or late cessation. EMR meta-data as a marker of acceptability will include proportion who decline participation, deviation from trial protocol and time to randomisation.

NVivo software will be used to code interviews into the 7 Theoretical Framework of Acceptability (TFA) using NVivo software. Thematic analysis, employing both deductive and inductive processes will be used to analyse the data.

For the economic analysis, the within-hospital costs assigned to each FN episode and separated into individual cost buckets will be collected and compared for the two interventions. This information is readily accessible from hospital information systems. Cost-effectiveness of the intervention will be presented as a cost-minimisation analysis to show the relative impact on costs of the STOP and CONTINUE arms.

<u>Sample size.</u> Small focus group sessions (4-5 participants) will be run until a target sample size of between 20-30 per group is achieved. This sample size has been recommended to reach data saturation in qualitative research.[27]

Recruitment and consent. The Consent process will follow that outlined in the main protocol – Section 4.7. Briefly, participants will be contacted in person (ie. for patients and families

admitted to hospital for FN care) and via email (for clinicians caring for oncology patients) and provided with the PICF. Where a participant agrees to a participate, a signed version will be returned via email or in person prior to interviews commencing. For clinicians, a follow-up email will be sent after two weeks to allow further expressions of interest. Participants have the right to withdraw prior or after the interview commencing.

Data storage. The discussion will be digitally recorded using MCRI-allocated Zoom and the audio file stored on MCRI servers under password protection and accessible in accordance with the Data Management Plan (see 9.2). Recording and transcription will be deleted at the conclusion of the research project and after a minimum of five years. Participants will be de-identified during hand transcription by the researcher and in any subsequent analysis or publication. Where a participant withdraws after the interview has been recorded, there comments will be excluded from hand transcription and analysis.

19.6 APPENDIX 6: ELSA-FN Trial video information script

Dr Gabrielle Haeusler
Dr Diane Hanna

Dr Gabrielle Haeusler (Consultant paediatric infectious diseases physician):
Hi my name is Gab Haeusler. I'm an infection specialist and researcher at the Royal Children's Hospital Melbourne. I'd like to tell you about a research study called ELSA FN. This stands for Early versus Late Stopping of Antibiotics for Febrile neutropenia.

[*Animation showing abbreviation*]

This study hopes will answer a really important question- how long should children and young people with febrile neutropenia get antibiotics for?

Febrile neutropenia is when you have a fever and low white blood cells. White cells are infection fighting cells. Febrile neutropenia happens often with cancer treatment and stem cell transplant and it's a common reason for kids to be admitted to hospital. Some people with febrile neutropenia have a bacterial infection requiring antibiotics but 2 in 3 people will have no infection and fever only.

[*Animation showing 2 in 3 no infection*]

There are two different approaches to antibiotics in febrile neutropenia. The first is giving antibiotics until the white cells start to rise. This generally means more antibiotics and maybe more time in hospital. You may have experienced that this is often the approach at Royal Children's Hospital.

The second approach is early stopping or short-course antibiotics. This means that antibiotics are stopped after 48 hrs if the patient is well and no infection is found. Studies in adults with cancer prove this is not only safe but it reduces the amount of antibiotics people get.

Giving less antibiotics may be a good thing. While antibiotics are important for killing bacterial infections they also have some side effects. People can feel sick, have diarrhoea or have kidney problems when antibiotics are given. One of the biggest worries though is development of resistant bacteria. These bacteria are stronger and tougher to treat, requiring special antibiotics. Giving too many antibiotics has caused more resistant bacteria worldwide.

Dr Diane Hanna (Consultant paediatric oncologist):
Hi my name is Di Hann and I'm a cancer specialist and researcher at the Royal Children's Hospital.

The ELSA FN study has been developed with all the cancer specialist here, including your doctor, and the ethics committee. This means that it is safe.

[*Animation two arms of trial and coin toss*]

ELSA FN compares short-course antibiotics and the standard longer-course antibiotics to see if one approach is better for patients and families. Short-course antibiotics is already standard practice for children with febrile neutropenia in some other paediatric cancer centres.

Because this is a randomised trial, the group kids end up in will be decided by chance, similar to tossing a coin. You have equal chance of being in either group but the computer will decide which group you are in. Once we know the group, we will tell you.

The choice to be in the trial is entirely up to patients and their families. If you agree to be in the trial, it will not interrupt your cancer treatment. If you are in the group where antibiotics are stopped early and you have another fever or get unwell, antibiotics will be started again without delay.

The information we need to answer this important research question is already being collected, for example your temperature and how long you are admitted to hospital for. If you want to be part of this study there are no extra blood tests or follow up visits. You will stay in hospital for monitoring until your white blood cells start to improve and the medical team are happy you are getting better.

Thank you for learning about ELSA FN and clinical research. We look forward to sharing results of the trial when they are ready. If you would like to be in the trial, chat with your medical team or one of the researchers.

[*Email address shown*]

19.7 APPENDIX 7: Trial poster (text)

Early versus Late Stopping of Antibiotics in Febrile Neutropenia (ELSA-FN) is recruiting children and young people who have a diagnosis of acute myeloid leukaemia (AML), acute

lymphoblastic leukaemia (ALL), lymphoma, or are planned for a haematopoetic stem cell transplant (HSCT).

This is a clinical trial in children with febrile neutropenia (fever and low white blood cells) looking at the safety of stopping antibiotics when patients are well and without fever for at least 48 hrs.

Learn more about ELSA FN by watching the information video below or ask your treating medical team for more information.

19.8 APPENDIX 8: Specimens for biobanking - completed biobank registration form

N/A
