# Supplementary material for: Protocol for an embedded randomised controlled trial of Early versus Late Stopping of Antibiotics in children with Febrile Neutropenia (ELSA-FN)
Source: PLoS One. 2024 Dec 9;19(12):e0311523. doi: 10.1371/journal.pone.0311523 (PMC11627426; doi:10.1371/journal.pone.0311523)
Supplement: S1 Table — (DOCX) [file pone.0311523.s003.docx]

| **Table 1.** Clinical definitions and electronic medical record translation | | |
| --- | --- | --- |
|  | **Clinical definition** | **EMR definition** |
| **Fever** | - Single temperature ≥ 38.0 °C^1^ | - Single temperature ≥ 38.0 °C |
| **Neutropenia** | ANC <500 cells/mm^3^ | ANC <500 cells/mm^3^or total WCC <1 x 10^9^/L |
| **Febrile neutropenia**  **onset** | First onset of fever in the setting of neutropenia | Fever within 24h of recorded neutropenia (pre- or post-fever) or recorded triage time if presenting to the emergency department with FN |
| **High risk febrile neutropenia** | FN occurring in patients at high risk of infection or adverse outcome including patients with diagnosis of AML; ALL induction, consolidation of delayed intensification phases; lymphoma in induction; on TOT17 protocol; or in first 100 days of HCT | FN occurring in patients with any of:   - AML (any phase) - ALL (induction/re-induction, intensification or consolidation treatment phase) - Lymphoma (induction) - ALL or LLy receiving TOT17 (induction, consolidation or continuation) - HCT day +30 |
| **End of febrile neutropenia episode** | Afebrile for >48h, no clinical instability for >48h and recovery of ANC to ≥500 cells/mm^3^ | No fever recorded for >48h and ANC documented ≥500 cells/mm^3^ |
| **Positive blood culture prior to randomisation** | A blood culture that is taken after FN onset that returns a positive result prior to randomisation | A blood culture that is taken after FN onset that becomes culture-positive with any organism prior to randomisation |
| **Admission to ICU prior to randomisation** | Admission to ICU for any duration after FN onset and prior to randomisation | Not admitted to ICU at the time of randomisation |
| **Admission to ICU for organ support** | Admission to ICU in which management includes any of the following:   - Inotropes/vasopressors - Renal replacement therapy - Invasive or non-invasive ventilation | Admission to ICU in which management includes any of the following:   - Inotropes/vasopressors: adrenaline, noradrenaline, dopamine, dobutamine, vasopressin, milrinone - Renal replacement therapy: continuous renal replacement therapy, intermittent haemodialysis, peritoneal dialysis - Invasive ventilation: mechanical ventilation - Non-invasive ventilation: bi-level positive airway pressure (BiPAP), continuous positive airway pressure |
| **Clinical Stability** | Physiologically stable at the time of randomisation. | -No MET call note within previous 48h (conscious state, blood pressure, heart rate, respiratory rate, respiratory effort or oxygen saturation have not breached age-based MET criteria) AND  -Not meeting clinical review criteria for heart rate within previous 48h (heart rate >95th percentile for age) AND  -Not in clinical review criteria (conscious state, blood pressure, heart rate, respiratory rate or oxygen saturation) at the time of randomisation[31] |
| **New fever episode** | A new fever after an afebrile period of >48h | A new fever after an afebrile (temperature <38°C) period of >48h |
| **New febrile neutropenia episode** | A new fever occurring during a new episode of neutropenia (ANC <500 cells/mm^3^) and more than 28 days after last randomisation | Neutropenia and a new fever occurring after the ‘end of FN episode’ and more than 28 days after last randomisation |
| **Prolonged febrile neutropenia** | Neutropenia and documented daily temperature ≥38.0°C for ≥5 days | Neutropenia and documented temperature ≥38.0°C every day for ≥5 days |
| **Unfavourable clinical course** | Any of the following occurring after randomisation and during the same period of neutropenia (ANC <500 cells/mm^3^): | |
|  | Recurrence of fever ≥38.0 °C after afebrile period of >48h | Documented temperature ≥38.0 °C after >48h period of temperature <38.0°C |
|  | Clinical instability | -One or more vital signs (conscious state, blood pressure, heart rate, respiratory rate or oxygen saturation) in age-based MET criteria, OR  -Two or more vital signs (blood pressure, heart rate, respiratory rate or oxygen saturation) simultaneously (+/- 4h) in age-based clinical review criteria[31] |
|  | - Admission to ICU | Admission to ICU for any reason |
|  | - New positive blood culture | Blood culture collected after randomisation and during same period of neutropenia that becomes culture-positive (with any organism) |
|  | - Death | Death (from any cause) |
| **Antibiotic duration** | - Duration of all antibiotics (excluding PJP and FN prophylaxis) and calculated as days of therapy | Date time of first dose antibiotic to date and time of last antibiotic dose (any antibiotic with the exception of prophylaxis using TMP-SMX, ciprofloxacin or levofloxacin) |
| **Total in-hospital LOS** | - Duration of in-hospital length of stay calculated from in-hospital admission date and time to discharge or transfer from in-hospital ward | Date and time of in-hospital admission to date and time of discharge or transfer from in-hospital ward |
| **Total hospital LOS** | - Duration of in-hospital length of stay calculated from randomisation date and time to discharge from hospital ward or HITH | Date and time of randomisation to date and time of hospital discharge (including from HITH) |
| **Unplanned readmission** | - Readmission to hospital within 28 days of randomisation | Unplanned readmission to hospital (inpatient ward) within 28 days of randomisation |
| **Duration of neutropenia** | - Days of neutropenia from FN onset to recovery of ANC ≥500 cells/mm^3^ | Date and time of fever onset to date and time of first ANC ≥500 cells/mm^3^ |
| **Cause of fever** | - Microbiologically defined infection (MDI): an infection that is clinically detectable and microbiologically proven. - Bacteraemia: a recognised pathogen cultured from one or more blood cultures (includes viridans group streptococci in the setting of concomitant mucosal barrier injury). Common commensals should be cultured from two or more blood cultures drawn on separate occasions. Where only a single blood culture is taken, and in the presence of a long term vascular catheter, common commensals cultured once may be included if an alternative source of infection is not identified. (Blood cultures drawn from different sites including different venepuncture or different lumens of the same central line, should undergo separate decontamination and are therefore considered drawn on “separate occasions”)[41] - Clinically defined infection (CDI): an infection that is clinically detectable but no MDI is identified - Fever without a focus: fever without a documented MDI or CDI | Will require manual allocation following chart review |
| **Time to first dose antibiotic** | - Time to first dose antibiotic calculated for initial FN episode and clinical failure (starting new antibiotic) | Date and time from first fever and/or clinical instability to first dose antibiotic |
| **28 days mortality** | - Death within 28 days of randomisation | Death within 28 days of randomisation |
| **Prescribed antimicrobial prophylaxis** | - Prescribed antimicrobial prophylaxis in 7 days prior to randomisation and/or within 28 days of randomisation | Any of the following prescribed in 7 days prior to randomisation and/or within 28 days of randomisation:  ciprofloxacin, levofloxacin, TMP-SMX, dapsone, pentamidine, amphotericin B, liposomal amphotericin, fluconazole, posaconazole, voriconazole, itraconazole, micafungin, caspofungin |
| **Antibiotic resistant infection or colonisation** | Antibiotic resistant infection or colonisation within 28 days of randomisation | Any of the following identified from sterile site culture (blood, urine, cerebrospinal fluid, peritoneal fluid, synovial fluid) or screening swab/stool:   - Methicillin-Resistant *Staphylococcus aureus* (MRSA): Staphylococcus aureus reported resistant to oxacillin - Extended Spectrum Beta Lactamase-producing enterobacterales (ESBL): *Escherichia coli, Klebsiella species., Enterobacter spp., Morganella spp., Providencia spp*. or *Proteus spp.* in which a transmissible ESBL enzyme or plasmid-mediated AmpC has been reported. - Carbapenemase-Producing Enterobacterales (CPE): *Escherichia coli, Klebsiella species., Enterobacter spp., Morganella spp., Providencia spp*. or *Proteus spp.* reported as resistant to meropenem - Vancomycin Resistant Enterococci (VRE): *Enterococcus faecalis* or *Enterococcus faecium* reported resistant to vancomycin. - Multidrug-resistant Pseudomonas aeruginosa (MRPAER): *P. aeruginosa* resistant to at least 2 or more of gentamicin, ciprofloxacin or a beta-lactams (e.g. piperacillin, ceftriaxone, ceftazidime, meropenem) |
| **Clostridium difficile infection** | 1. Diarrhoea (usually defined as 3 or more loose stools in a 24-hour period), ileus, toxic megacolon or pseudomembranous colitis (identified by colonoscopy) with positive stool test for toxin-producing *C. difficile* | Positive laboratory test result for *C. difficile*toxin A and /or B tested on an unformed stool specimen |
| **Clinically documented infection (CDI)** | 1. An infection that is clinically diagnosed but no pathogen cultured | Will require manual allocation following chart review |
| ANC, absolute neutrophil count; WCC, white cell count; h, hours; FN, febrile neutropenia; AML, acute myeloid leukaemia; ALL, acute lymphoblastic leukaemia; LLy, lymphoblastic lymphoma; TOT17, total therapy XVII for newly diagnosed patients with acute lymphoblastic leukaemia and lymphoma; HCT, haematopoietic cell transplant; ICU, Intensive Care Unit; MET, medical emergency team; TMP-SMX, trimethoprim-sulfamethoxazole; PJP, Pneumocystis jirovecii pneumonia; HITH, hospital in the home | | |

**Reference**

1. Haeusler GM, Phillips RS, Lehrnbecher T, Thursky KA, Sung L, Ammann RA. Core outcomes and definitions for pediatric fever and neutropenia research: a consensus statement from an international panel. Pediatr Blood Cancer 2015;62:483–9. https://doi.org/10.1002/pbc.25335.
